# R-Spondin1 Regulates Fate of Enteric Neural Progenitors via Differential LGR4/5/6 Expression in Mice and Humans

**DOI:** 10.1016/j.jcmgh.2025.101642

**Published:** 2025-09-24

**Authors:** Melanie Scharr, Simon Scherer, Jörg Fuchs, Bernhard Hirt, Peter H. Neckel

**Affiliations:** 1 Institute of Clinical Anatomy and Cell Analysis, University of Tübingen, Tübingen, Germany; 2 Department of Pediatric Surgery, University Children’s Hospital Tübingen, Germany

**Keywords:** Enteric Nervous System, LGR4/5/6, Neurogenesis, R-Spondin, WNT signaling

## Abstract

**Background & Aims:**

Regeneration and cytodifferentiation of various adult epithelial stem cell compartments are controlled by the WNT agonist R-Spondin1 (RSPO1) and the Leucin-rich-repeat-containing G-protein-coupled receptors (LGR4/5/6). We hypothesized that RSPO1-LGR signaling is also involved in regulating neuroregeneration and homeostasis of the postnatal enteric nervous system (ENS).

**Methods:**

We isolated neural crest-derived ENS cells from wnt1-tomato mice and patient samples, which were evaluated using pharmacological in vitro studies under RSPO1 stimulation. We use proliferation assays (BrdU incorporation, Ki67 staining), as well as neuronal differentiation screenings. We performed fluorescence-activated cell sorting-based in vitro assays to stratify human ENS cells for LGR receptor expression, and to characterize them by immunofluorescence colabeling studies in vivo.

**Results:**

If applied to murine and human ENS progenitors, RSPO1 led to an increased proliferation (*P* = .002), followed by enhanced enteric neurogenesis (*P* < .001). This coincided with an upregulation of LGR4 expression during ENS progenitor proliferation (*P ≤* .001) in vitro. In contrast, we observed a reduced proliferation in ENS progenitors expressing LGR5 (*P* ≤ .001), whereas LGR6 was not expressed by proliferative ENS progenitors (*P* ≤ .05). Instead, LGR5 and LGR6 expression increased over the course of induced neuronal differentiation (LGR5: *P* ≤ .001 and LGR6: *P* ≤ .05), consistent with the in vivo expression.

**Conclusions:**

LGR receptor expression therefore might represent a previously unknown mechanism influencing the fate decision of ENS progenitor cells between proliferation and neuronal differentiation. Thus, our study is essential for our understanding of regenerative aspects of the postnatal ENS in health and disease.

**Summary**

Our study shows that the WNT signaling agonist R-Spondin1 drives fate decision between neural proliferation and neuronal differentiation via differential Leucin-rich-repeat-containing G-protein-coupled-receptor expression of postnatal murine and human enteric nervous system progenitors.

Neurons and glial cells of the enteric nervous system (ENS) are constantly exposed to an ever-changing environment, including motility patterns,^1^ microbiome,^2^ or immunological interactions.^3^ The ENS needs to adapt to these fluctuations to maintain its cellular homeostasis. Generally, tissue homeostasis involves self-renewal, characterized by cell proliferation, cytodifferentiation of stem and progenitors, and functional integration (reviewed in detail by Seifert et al^4^). In the murine ENS, neurogenesis is detectable within the first postnatal days.^5^^,^^6^ Additionally, refinements of the neurochemical coding and synaptic wiring continues until adolescence.^7^^,^^8^ However, whether proliferation is preserved throughout postnatal life is controversially discussed.^9^ So far, neurogenesis in the adult ENS has been convincingly described only under pathological/experimental settings like chemical denervation^10^ and colitis.^11^ Nevertheless, many studies demonstrated the isolation, expansion, and differentiation of neurogenic cells from the postnatal ENS of rodents and human patients.^12^, ^13^, ^14^ This suggests that under physiological conditions ENS progenitors remain quiescent in vivo, arguably due to the local microenvironment. This ENS progenitor niche remains poorly characterized^15^ and requires explorative in vitro studies.

In the intestinal epithelium, the interplay between cycling intestinal stem cells (ISCs),^16^ quiescent stem cells,^17^ and dedifferentiating cell types^18^ enabling self-renewal and regeneration, is well-described. The hierarchical differentiation processes from ISCs to mature functional cells are tightly controlled by cell-cell-communication systems and secreted factors from the epithelium and the surrounding mesenchyme.^19^ Among those cell-cell communication system, the β-catenin-dependent (canonical) WNT signaling pathway is well-characterized and forms a gradient with decreasing activity along the crypt-villus axis.^20^ Thereby, the WNT pathway is a key driver for proliferation, differentiation, and survival of ISCs located at the base of the intestinal crypts.^21^ Further, the WNT agonists R-Spondin1-4 (RSPO1-4) and their receptors, the Leucin-rich-repeat-containing G-protein-coupled-receptors (LGR4/5/6), are crucial to maintain the proliferative capacity of ISCs.^22^^,^^23^ Interestingly, depending on the maturation stage of the intestinal epithelium, LGR4 and LGR5 receptors serve different functions maintaining active and quiescent stem cells^21^ and can also be used as markers to generate intestinal organoids from the mouse intestinal epithelium.^24^^,^^25^ Whether LGR4/5/6 receptors play comparable roles in ENS homeostasis is not known.

Interestingly, diverse canonical and β-catenin-independent (noncanonical) WNT signaling components are expressed during ENS development.^26^, ^27^, ^28^ Moreover, dysregulation in WNT signaling might contribute to the pathogenesis of enteric neuropathies.^29^ Still, their impact on proliferation and differentiation of postnatal ENS progenitors in vivo has not been thoroughly explored. In this context, previous work of our group demonstrated that canonical WNT signaling is inactivated during the transition from proliferation to differentiation of postnatal ENS progenitor cells in vitro.^30^ In addition, pharmacological activation of canonical WNT signaling by WNT3A increases the proliferative capacity of postnatal ENS progenitors from rodent models and human infants in vitro, leading to a higher yield of newly generated neurons.^31^ Moreover, we have recently shown that several WNT ligands and receptors as well as RSPO1-4 and LGR4/5/6 receptors are expressed on mRNA level in ENS cells in adult mice in vivo.^32^

Interestingly, Stavely and colleagues demonstrated that enteric mesenchymal cells (EMCs) shape the fate of postnatal ENS progenitors by secreting morphogens including Wnt ligands, as well as RSPO1 and RSPO3, with corresponding receptors expressed on ENS progenitors like LGR4.^33^ The present study evaluates the cell biological function of RSPO1-LGR4/5/6 signaling in the postnatal ENS. We found that RSPO1 influences fate decision between proliferation and neuronal differentiation in murine and human ENS progenitors, possibly due to differential expression of LGR4/5/6 receptors. Our data sets previous in vivo studies on ENS regeneration into a new context and paves the way for a mechanistic understanding of activating quiescent ENS progenitors in the postnatal ENS.

## Results

### RSPO1 Stimulation Has a Pro-proliferative Effect on Postnatal ENS Progenitors

RSPO-LGR interaction enhances active WNT signaling by inhibition of the WNT-receptor-degrading ligases RNF43 and ZNRF3.^34^ Hence, increasing canonical^35^ and noncanonical WNT signaling activity^36^ by elevating the concentration of Frizzled (FZD) family WNT-receptors and the coreceptors LRP5/6 on the cell-surface.

We evaluated postnatal ENS progenitors for RSPO ligand and LGR receptor expression using enterospheres of newborn (P0) C57BL6/J mice. In brief, ENS progenitors were isolated from the Tunica muscularis of newborn pups, as previously described.^37^^,^^38^ The culture of murine Tunica muscularis cells (including ENS progenitors) under proliferation conditions resulted in 3-dimensional spheroids, termed enterospheres.

**Figure 1:**
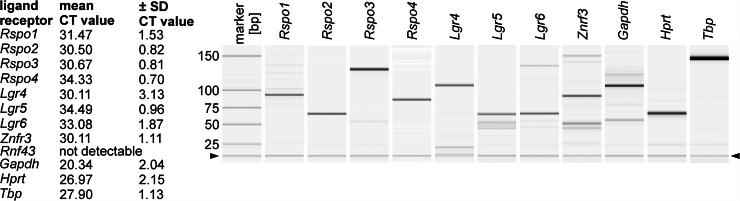
**RSPO ligands and LGR receptors are expressed in murine ENS progenitors.** Identification of RSPO ligands *Rspo1-Rspo4*, RSPO receptor *Lgr4, Lgr5, and Lgr6,* as well as the transmembrane ligase *Znrf3* in 5-days-old enterospheres by qRT-PCR (CT-values expressed as mean ± SD) and by capillary electrophoresis (*arrowhead* indicates position of alignment marker).

After culturing for 5 days in vitro (5 div), we detected a robust mRNA expression of the ligands *Rspo1-4* and *Lgr4-6,* as well as *Znrf3*, using reverse transcription-polymerase chain reaction (RT-PCR) (Figure 1). Together with previous studies on *Fzd-* and *Lrp5/6*-receptors expression in murine enterospheres,^31^^,^^32^^,^^37^ this indicates that our in vitro model is suitable for pharmacological probing with RSPO1.

**Figure 2:**
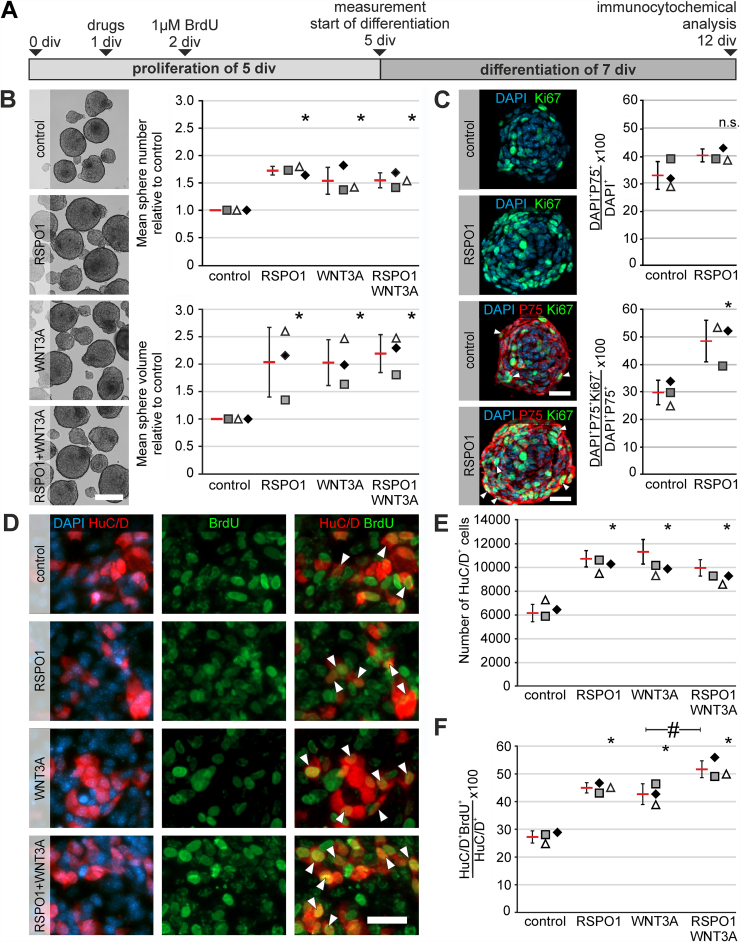
**Pro-proliferative effect of RSPO1 increases enteric neurogenesis in mice.** (*A*) Enterospheres were cultured for 12 div. RSPO1 and/or WNT3A was applied after 1 div, BrdU after 2 div. (*B*) representative enterosphere cultures after 5 div. Scale: 100 μm. RSPO1, Wnt3A, and RSPO1 + Wnt3A increased the number and cumulative volume of enterospheres compared with control (ANOVA, Fisher LSD, bars and error bars are mean ± SD; n = 3). (*C*) Micrographs display immunofluorescence colabeling studies with Ki67 (*green*) and P75 (*red*) and the nuclear marker DAPI (*blue*), on paraffin sections of enterospheres that were cultured for 5 days in vitro (5 div) under proliferative conditions. RSPO1 was applied after 1 div. Untreated enterospheres served as control. Scale bar: 50 μm. The dot plots indicate the percentage of P75^+^DAPI^+^ and proliferative P75^+^Ki67^+^DAPI^+^ cells (mean ± SD) for the control and RSPO1-stimulated group. Asterisk indicates significant differences in comparison to the control group. Data points for independent biological replicates are represented by different symbols. RSPO1 stimulation had no significant effect on P75^+^DAPI^+^ cells (ANOVA, Fisher LSD post-hoc test, mean ± SD; n = 3), yet increased the number of proliferative Ki67^+^P75^+^DAPI^+^ cells (ANOVA, Fisher LSD, mean ± SD; n = 3). Source data are provided as a Source data file. (*D*) Immunostaining for BrdU and HuC/D after 12 div. BrdU^+^HuC/D^+^ neurons were detected in all groups (*arrowheads*). Scale: 40 μm. (*E*) Number of HuC/D^+^ neurons increased after RSPO1 stimulation compared with control. (*F*) RSPO1 stimulation increased the number of BrdU^+^HuC/D^+^ cells compared with control. RSPO1 + WNT3A stimulation had an additional significant effect to WNT3A stimulation. Asterisks indicate significant differences compared with controls in *C, E, and F* (ANOVA, Fisher LSD, bars and error bars are mean ± SD; n = 3). Source data are provided as a Source data file.

To evaluate the cell biological function of RSPO1, ENS progenitors from P0 C57BL6/J mice were expanded 5 days and treated with RSPO1 (100 ng/mL), WNT3A (20 ng/mL) alone or in combination after 1 div. Untreated cultures served as controls. After 5 div, the number (analysis of variance [ANOVA], Fisher LSD post-hoc test, mean fold change ± standard deviation [SD]: RSPO1: 1.53 ± 0.25 [*P* = .002]; WNT3A: 1.70 ± 0.08 [*P* ≤ .001]; Combination: 1.53 ± 0.14 [*P* = .002]; n = 3) and cumulative volume (ANOVA, Fisher LSD post-hoc test, mean fold change ± SD: RSPO1: 2.29 ± 0.42 [*P* = .005]; WNT3A: 2.30 ± 0.64 [*P* = .005]; Combination: 2.46 ± 0.35 [*P* = .003]; n = 3) of enterospheres in all treated groups increased compared with control (Figure 2*A–B*).

This suggests a stimulation of proliferation in ENS progenitors by RSPO1. Within the Tunica muscularis and hence within our spheroid cultures, only neural cells, including neural progenitor cells, express P75. Therefore, we evaluated the proliferative rate of P75^+^ neural cells after RSPO1 stimulation using Ki67 staining. The percentage of P75^+^ cells barely changed after RSPO1 stimulation compared with controls (ANOVA, Fisher LSD post-hoc test, mean ± SD: Control: 33.0% ± 5.06%; RSPO1: 39.9% ± 2.31%; n = 3; *P* = .098) (Figure 2*C*). However, the percentage of actively cycling Ki67^+^P75^+^ cells significantly increased after RSPO1 stimulation by 1.68-fold compared with control, suggesting that among all neural cells a subpopulation proliferates in response to RSPO1 stimulation (ANOVA, Fisher LSD post-hoc test, mean ± SD: Control: 28.7% ± 5.58%; RSPO1: 48.1% ± 7.55%; n = 3; *P* = .023) (Figure 2*C*).

To analyze whether RSPO1 stimulation induces neurogenesis, stimulated enterospheres were differentiated for 7 div and stained for the pan-neuronal marker HuC/D (Figure 2*D*). The number of HuC/D^+^ neurons increased by 1.59-fold after RSPO1 stimulation compared with control. This effect was comparable to a WNT3A-stimulus (ANOVA, Fisher LSD post-hoc test, mean ± SD: Control: 6382 ± 733; RSPO1: 10,117 ± 1035 *[P* < .001]; WNT3A: 10,229 ± 688 [*P* < .001], Combination: 9220 ± 694 (*P* < .002); n = 3) (Figure 2*E*). Additionally, BrdU incorporation revealed new-born BrdU^+^HuC/D^+^ neurons in all experimental groups, suggesting that these cells derived from proliferating ENS progenitors after isolation (Figure 2*D*). RSPO1 stimulation increased the percentage of newborn BrdU^+^HuC/D^+^ neurons by 1.65-fold compared with control. Further, we observed an additional significant effect of RSPO1 + WNT3A (1.99-fold compared with control), (ANOVA, Fisher LSD post-hoc test, mean ± SD: Control: 27.2% ± 2.20%; RSPO1: 45.0% ± 1.83% [*P* < .001]; WNT3A: 42.7% ± 3.75% [*P* < .001], Combination: 51.6% ± 3.73% [*P* < .001]; n = 3) (Figure 2*F*).

Therefore, RSPO1 has a pro-proliferative effect on neural cells, which is further reflected in a higher yield of newborn BrdU^+^HuC/D^+^ neurons in vitro.

### RSPO1-mediated Effect Acts Directly on ENS Progenitors

**Figure 3:**
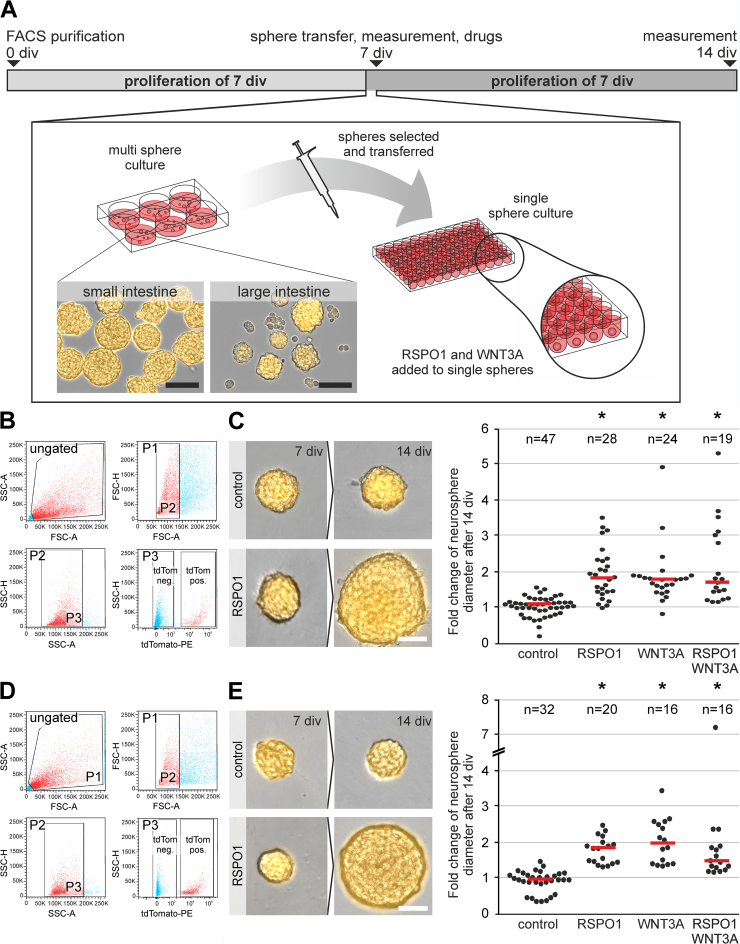
**RSPO1 effect acts directly on murine ENS cells.** (*A*) FACS-purified ENS progenitors of small and large intestine were cultured for 7 div as a multisphere culture. Afterwards, equally sized neurospheres were transferred into single sphere culture until 14 div. Brightfield images were taken before RSPO1 and/or WNT3A were added and after 14 div. Scale: 100 μm. (*B and D*) Representative FACS of tdTomato expressing ENS cells from small (*B*) and large (*D*) intestinal samples derived from postnatal day 60 old Wnt1Cre2 mice. The scatter blots represent the gating strategy (ungated, P1–P3) for tdTomato negative (P4) and tdTomato positive (P5) cell pools derived from small and large intestine. Each population that underwent further gating are colored in *red*. All events counted were first gated as P1 to exclude dead cells and debris, as these could be found at the bottom left corner of the first dot blot of each sample. Next, P1 was gated regarding its properties in forward (P2) and sideward (P3) scatter mode to exclude doublets and aggregates. Finally, P3 was gated regarding its tdTomato-expressing property. (*C and E*) Representative images of the same tdTomato-positive neurosphere after 7 div and 14 div under control and RSPO1-treated condition from small (*C*) and large (*E*) intestinal samples. Scale: 40 μm. Dot plots depicts the fold change of neurosphere diameter after 14 div from small (*C*) and large (*E*) intestine samples. Each dot represents one neurosphere; *red bars* represent the median. RSPO1 and WNT3A stimulation significantly increased neurosphere diameter compared with control (ANOVA on Ranks, Dunn’s method, bars indicate the median fold change; 4 biological replicates).

To exclude additive effects by WNT-ligand secreting mesenchymal cells,^33^ and to assess if RSPO1 acts directly on neural cells, we cultured fluorescence-activated cell sorting (FACS)-purified ENS cells using 2-month-old wnt1-tomato mice (Figure 3*A*). In brief, FACS-purified ENS cells (Figure 3*B and D*), both derived from the small and large intestine, were cultured 7 div under proliferative conditions. Then, similar-sized tomato-positive neurospheres were transferred to a 96-well-plate containing proliferation medium, and brightfield images were taken. Afterwards, neurospheres were stimulated with RSPO1 (100 ng/mL), WNT3A (20 ng/mL), or RSPO1 + WNT3A, and cultured for another 7 div. Brightfield images were taken after 14 div. Untreated spheres served as control. Additionally, the single-sphere cultures exclude fusion artefacts during proliferation.

Similar to the results obtained from unpurified enterosphere-cultures, we found an increase of small intestinal neurosphere size in all treated groups, whereas control spheroids largely stagnated (Figure 3*B–C*). Like WNT3A, RSPO1 -stimulation significantly increased neurosphere size (ANOVA on ranks, post-hoc: Dunn’s method, median fold change: control: 1.12; n = 47; RSPO1: 1.84; n = 28 [*P* ≤ .001]; WNT3A: 1.81; n = 24 [*P* ≤ .001], RSPO1 + WNT3A: 1.74; n = 19 [*P* ≤ .001], 4 biological replicates) (Figure 3*C*).

Comparable results were obtained from large intestinal neurospheres (ANOVA on Ranks, post-hoc: Dunn’s method, median fold change: control: 1.02; n = 32; RSPO1: 1.95; n = 20 [*P* ≤ .001]; WNT3A: 2.08; n = 16 [*P* ≤ .001], RSPO1 + WNT3A: 1.56; n = 16 [*P* ≤ .001]; 4 biological replicates) (Figure 3*D–E*).

**Figure 4:**
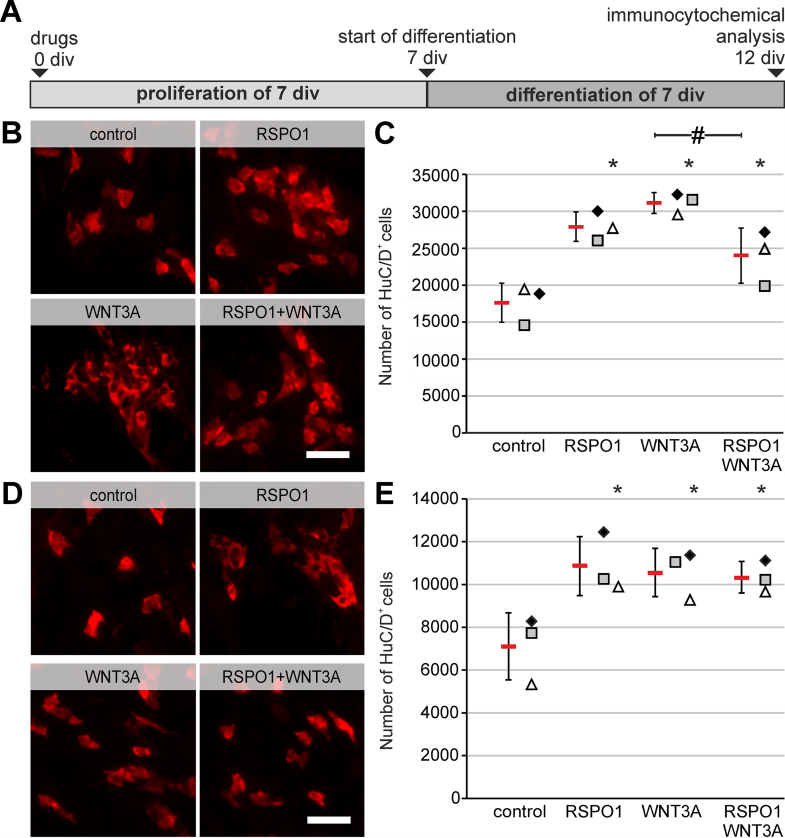
**RSPO1-mediated effect acts directly on small and large intestine derived ENS progenitors.** (*A*) FACS-purified ENS progenitors of small (*B–C*) and large (*D–E*) intestinal samples from 2-month-old (P60) mice were cultured for 7 days under proliferative conditions in vitro (7 div) as a multisphere culture. After 7 div, cells were cultured for 7 div under differentiation conditions and underwent immunocytochemical analysis for HuC/D. (*B and D*) Micrographs show representative images of HuC/D^+^ neurons (*red*) in all experimental groups after differentiation. Scale bar: 50 μm. (*C and E*) Displays the quantification of the number of HuC/D^+^ neurons. Data points for different experimental groups are represented by different symbols. Number of neurons increased after RSPO1 stimulation compared with untreated control in small as well as large intestine samples. Asterisk indicates significant differences compared with control. Source data are provided as a Source data file.

Further, we evaluated neurogenesis by quantifying the total amount of HuC/D^+^ neurons (Figure 4*A*). In cultures, both derived from small and large intestine, RSPO1-stimulation significantly increased the number of HuC/D^+^ cells compared with control control (small intestine: ANOVA, Fisher LSD post-hoc test, mean ± SD: control: 18,072 ± 2649; RSPO1: 27,933 ± 1995 [*P* = .002]; WNT3A: 31,131 ± 1412 [*P* < .001], RSPO1 + WNT3A: 23,996 ± 3731 [*P* = .023]; n = 3) (Figure 4*B–C*) (large intestine: ANOVA, Fisher LSD post-hoc test, mean ± SD: control: 7246 ± 1564; RSPO1: 10,844 ± 1375 *[P* = .007]; WNT3A: 10,543 ± 1126 [*P* < .012], RSPO1 + WNT3A: 10,319 ± 735 [*P* = .016]; n = 3) (Figure 4*D–E*). Interestingly, the combinatory treatment clearly showed less effect on FACS-purified ENS cells from small intestine compared with large intestine (Figure 4*C and E*). Overall, the described RSPO1 effect: (1) does not depend on WNT-secreting mesenchymal cells but could be further augmented; (2) RSPO1 acts directly on postnatal ENS progenitors; and (3) this effect is reproduceable on ENS progenitors isolated from later postnatal stages.

### RSPO1 Stimulates Proliferation and Neurogenesis in Human ENS Progenitors

**Figure 5:**
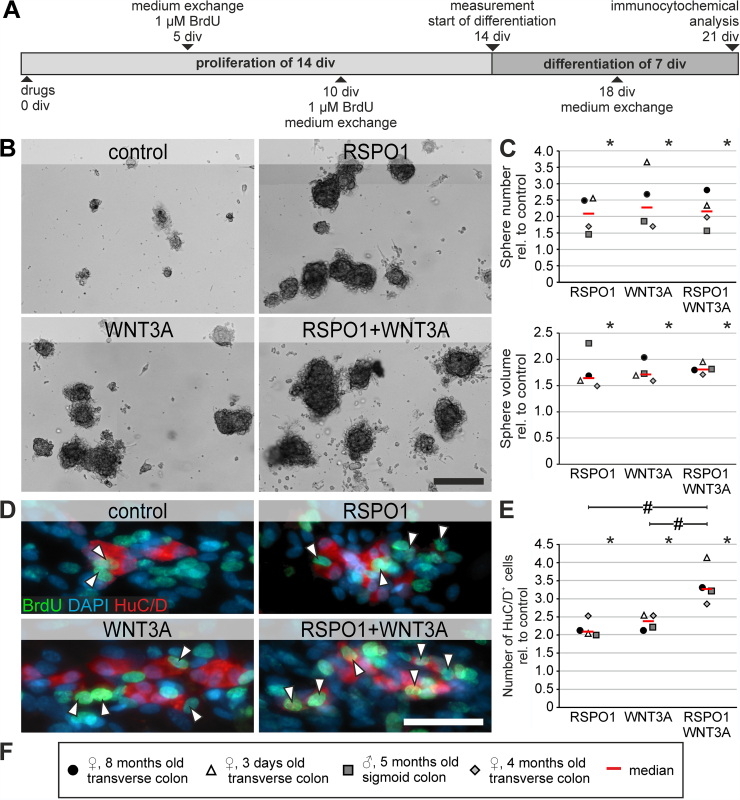
**Neurogenesis is increased in human ENS progenitors by RSPO1.** (*A*) Human *enterospheres* were cultured for 21 div. RSPO1 and/or WNT3A were added directly after seeding. BrdU was added after 5 and 10 div. (*B*) Representative human enterospheres after 14 div. Scale: 200 μm. (*C*) After 14 div, RSPO1 and/or WNT3A led to an increase in the number and cumulative volume of enterospheres relative to control. Data points for different patients are represented by different symbols. (*D*) BrdU-incorporation showed BrdU^+^HuC/D^+^ neurons in all groups (*arrowheads*). Scale: 50 μm. (*E*) The number of HuC/D^+^ neurons significantly increased after RSPO1 stimulation compared with control. Notable is the significant additional effect of RSPO1 with WNT3A. Asterisks indicate significant differences compared with controls (*C and E*); the hash indicates significant difference between groups (ANOVA on Ranks, Student-Newman-Keuls, median fold change [*red bar*]; n = 4). (*F*) Patient data of gut specimens used. Source data are provided as a Source data file.

To assess the clinical relevance of our findings, we evaluated the RSPO1 effect on ENS progenitors from 4 individual patients. Human enterospheres proliferated for 14 div; drugs were added directly after seeding. BrdU was added after 5 div and 10 div. After cell expansion, cells were differentiated for 1 week before HuC/D- and BrdU-immunolabeling (Figure 5*A and F*). After 14 div, the number (ANOVA on ranks, post-hoc: Student-Newman-Keuls Method, median fold change [red bar]: RSPO1: 2.09 [*P* = .028]; WNT3A: 2.27 [*P* = .028]; RSPO1 + WNT3A: 2.16 [*P* = .028]; n = 4) (Figure 5*B*) and volume (ANOVA on Ranks, post-hoc: Student-Newman-Keuls Method, median fold change [red bar]: RSPO1: 1.64 [*P* = .023]; WNT3A: 1.71 [*P* = .023]; RSPO1 + WNT3A: 1.81 [*P* = .023]; n = 4) (Figure 5*C*) of human enterospheres significantly increased in all treated groups compared with control. In addition, we found BrdU^+^HuC/D^+^ neurons in all groups, indicating proliferation in vitro (Figure 5*D*).

Moreover, RSPO1 stimulation increased the median number of HuC/D^+^ cells compared with control stimulation. In contrast to murine ENS progenitors, the combinatory treatment significantly increased the amount of HuC/D^+^ cells by 3.27-fold (ANOVA on Ranks, post-hoc: Student-Newman-Keuls Method, median fold change: RSPO1: 2.09 [*P* = .004]; WNT3A: 2.38 [*P* = .004); RSPO1 + WNT3A: 3.27 [*P* = .004]; n = 4) (Figure 5*E*). Thus, the RSPO1 effect was reproducible on human ENS progenitors, suggesting a conserved signaling mechanisms among species.

### LGR5 and LGR6 Receptors Are Expressed Mainly on Differentiated Enteric Neurons and Less in Glial Cells in the Human Gut

**Figure 6:**
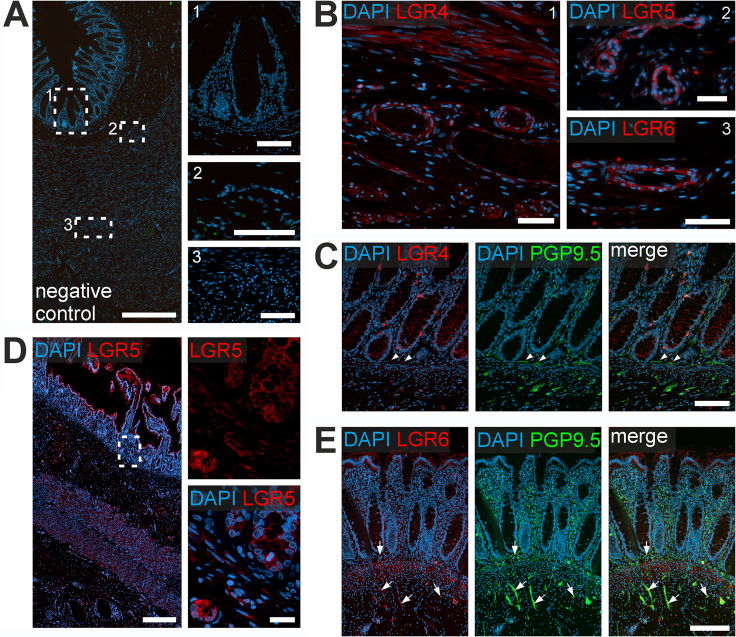
**LGR receptor expression in other compartments of small intestine.** (*A*) Displays an overview of a transversal section of the human small intestine section stained with secondary antibodies only as a negative control and the nuclear stain DAPI (*blue*). *White rectangles* indicate the location of the high-power magnification micrographs 1–3 on the right. Scale bars: overview 500 μm; details: 100 μm (1), 50 μm (2-3). (*B*) Shows representative images of blood vessels located in the Tela submucosa. LGR4 (1), LGR5 (2) and LGR6 (3), (*red*) are expressed in the Tunica media of arterioles. Further, we found a slight LGR4 expression in the Lamina muscularis mucosae. Nuclear marker DAPI (*blue*). Scale bars: 50 μm (B1-B2), 25 μm (B3). (*C*) Depicts a representative image of small intestine sample stained for LGR4 (*red*), the neuronal marker PGP9.5 (*green*), and the nuclear marker DAPI (*blue*). LGR4 was expressed throughout the crypt region until the villus border. Further, we detected LGR4 colocalization with PGP9.5-colabeled neurites surrounding the mucosal crypts (*arrowheads*). Scale bar: 100 μm. (*D*) Depicts small intestinal sample stained for LGR5 and the nuclear marker DAPI (*blue*). LGR5 expression was restricted to epithelial cells located at the crypt bottom (high power magnification). Scale bars: overview 600 μm; details: 20 μm. (*E*) Shows representative image of human small intestine stained for LGR6 (*red*), PGP9.5 (*green*), and the nuclear stain DAPI (*blue*). LGR6 was expressed throughout the Tunica mucosa predominantly in the Lamina muscularis mucosa. In addition, we detected some staining for LGR6 in PGP9.5-colabeled neurites outside the ganglia within the Tunica muscularis and in the fine neurite network surrounding the mucosal crypts (*arrow*). Scale bar: 100 μm.

**Figure 7:**
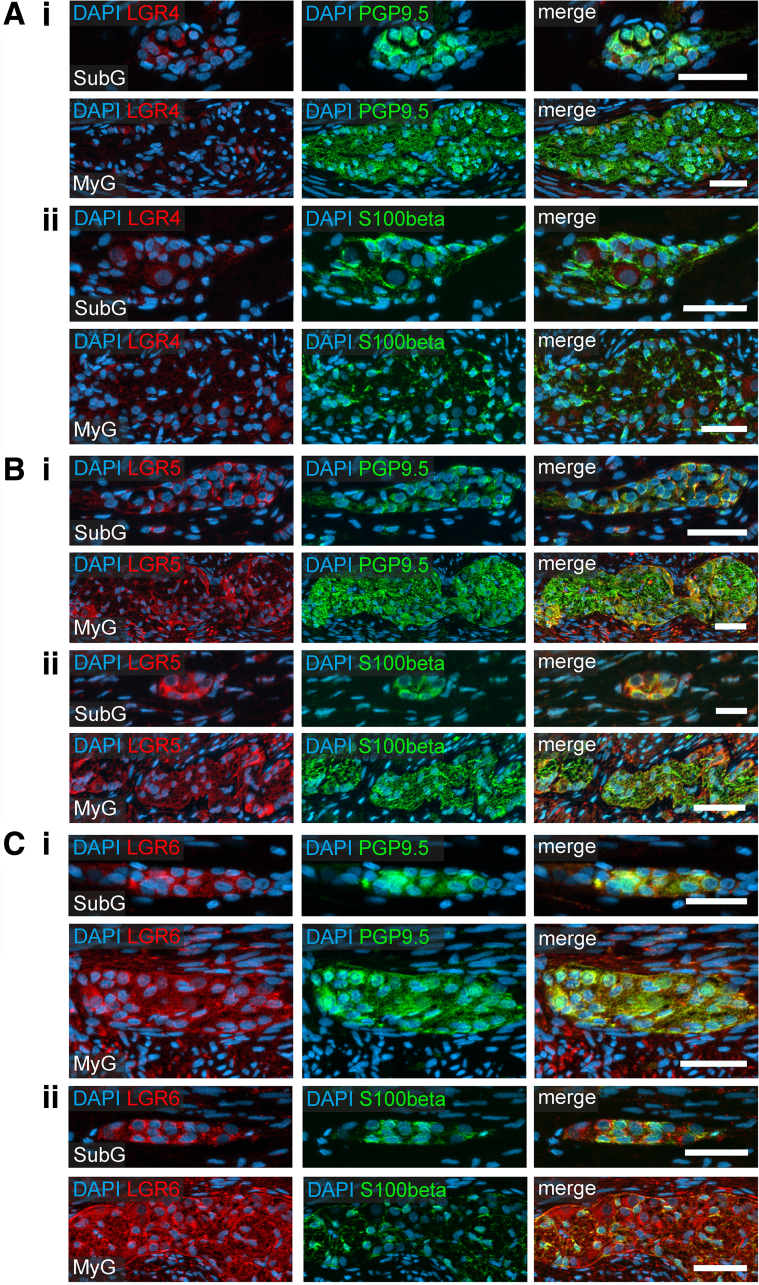
**Enteric neurons of human small intestine express the RSPO1 receptors LGR4, LGR5, and LGR6.** Immunohistochemical co-labeling studies of small intestinal resectates of pediatric patients for LGR4 (*A*), LGR5 (*B*), and LGR6 (*C*) (*red*); as well as the neuronal marker PGP9.5 (*green; i*), the glia cell marker S100beta (*green; ii*), and the nuclei marker DAPI (*blue*). Immunoreactivity for all 3 LGR receptors was found in submucosal and myenteric ganglia of small intestine (see also Table 1). In contrast to LGR4 staining intensity, LGR5 and LGR6 intensities were more intense in submucosal (SubG) and myenteric (MyG) ganglia. LGR5 and LGR6 expression was predominantly found in enteric neurons of submucosal (SubG) and myenteric (MyG) ganglia, whereas little to no immunoreactivity was observed for LGR4. Compared with enteric neurons, less signal intensity was detected in enteric glia cells of submucosal and myenteric ganglia. Scale bar: 50 μm.

We then analyzed the expression of LGR4/5/6-receptors in the human small and large intestine with immunhistochemistry. Although we detected LGR4/5/6-immunoreactivity in the ENS and other tissues of the gut wall (Figure 6 and 7; Table 1), we did not observe region-specific differences between small and large intestine. Our stainings confirmed LGR4/5/6-expression in smooth muscle cells in the Tunica muscularis and arterioles within the intestinal wall (Figure 6*A* for negative controls and Figure 6*B*). Moreover, LGR4 was expressed throughout the crypt region up to the villus border and slightly in the Lamina muscularis mucosae (Figure 6*C*). Further, we found a slight LGR4 immunoreactivity in PGP9.5-labeled neurites surrounding the mucosal crypts (Figure 6*C*, *arrowheads*). LGR5 expression was restricted to epithelial cells located at the crypt bottom (Figure 6*D*).

**Table 1:** Semiquantitative Evaluation of LGR Receptor Expression Within the Human Small and Large Intestine

Small intestine					
LGR	Submucous plexus (neuron/glia)	Myenteric plexus (neuron/glia)	Tunica muscularis (circular/longitudinal)	Crypts	Villus tip
LGR4	++/+	++/+	+/+	–	–
LGR5	++/+	++/+	+/+	+	–
LGR6	+++/+	+++/+	+/+	–	–

Large intestine					
LGR	Submucous plexus (neuron/glia)	Myenteric plexus (neuron/glia)	Tunica muscularis (circular/longitudinal)	Crypts	Epithelial surface
LGR4	++/+	++/+	+/+	–	–
LGR5	+++/+	+++/+	+/+	+	–
LGR6	+++/+	+++/+	+/+	–	++

–, no signal; +, low intensity; ++, medium intensity; +++, high intensity.

LGR6 was expressed throughout the Tunica mucosa predominantly in the Lamina muscularis mucosae (Figure 6*E*). Additionally, we detected LGR6 in PGP9.5-expressing neurites outside the ganglia within the Tunica muscularis and in the neurites surrounding mucosal crypts (Figure 6*E*, *arrows*).

**Figure 8:**
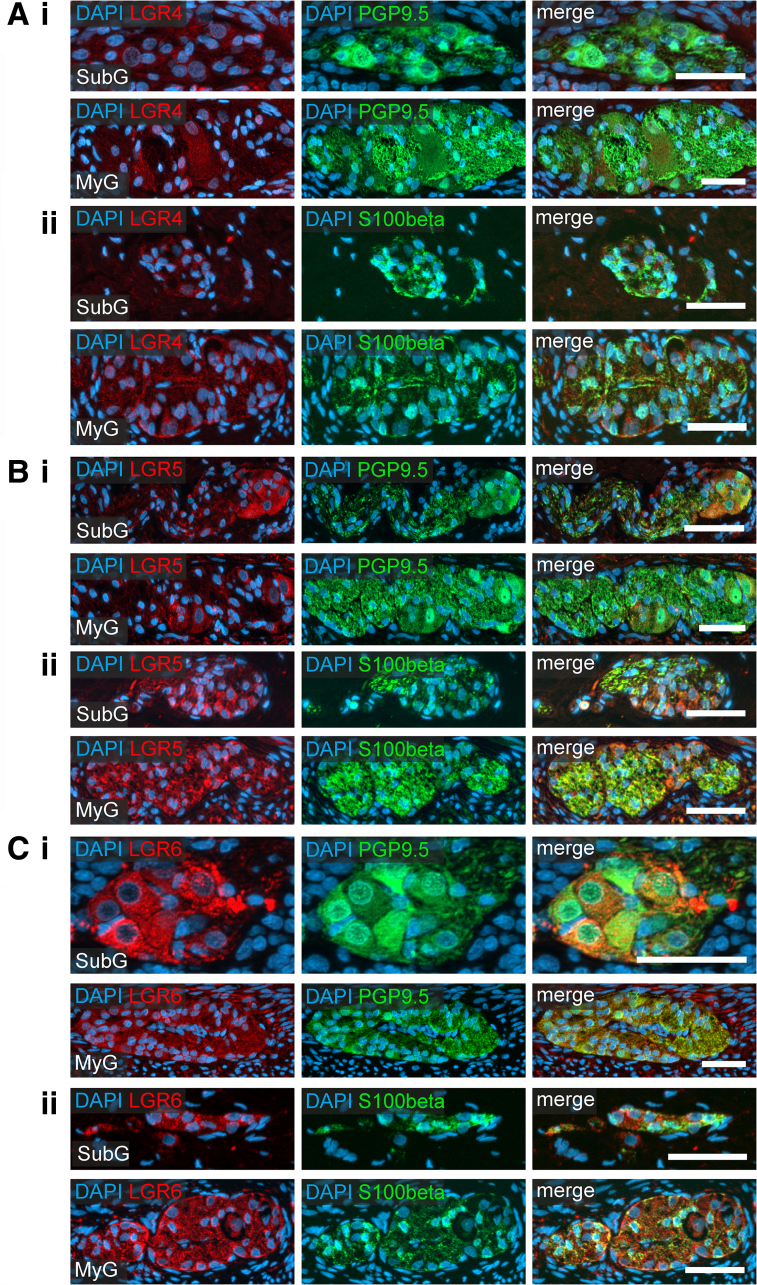
**RSPO1 receptors LGR4, LGR5, and LGR6 are expressed in large intestinal submucosal and myenteric neurons.** Immunohistochemical colabeling studies of large intestinal resectates of pediatric patients for LGR4 (*A*), LGR5 (*B*), and LGR6 (*C*) (*red*); as well as the neuronal marker PGP9.5 (*green; i*), the glia cell marker S100beta (*green; ii*), and the nuclei marker DAPI (*blue*). Immunoreactivity for all 3 LGR receptors was found in submucosal and myenteric ganglia of large intestine (see also Table 1). Compared with small intestine samples, the perceived signal intensity of each receptor was more intense. In contrast to LGR4 staining intensity, LGR5 and LGR6 intensities were more intense in submucosal (SubG) and myenteric (MyG) ganglia. LGR5 and LGR6 expression was predominantly found in enteric neurons of submucosal (SubG) and myenteric (MyG) ganglia, whereas little to no immunoreactivity was observed for LGR4. Compared with enteric neurons, less signal intensity was detected in enteric glia cells of submucosal and myenteric ganglia. Scale bar: 50 μm.

In the ENS, we found a weak homogeneous cytoplasmic LGR4 immunoreactivity in PGP9.5-labeled enteric neurons (Figures 7*Ai* and 8*Ai*) and in S100beta-labeled glial cells (Figure 7*Aii* and 8*Aii*) of submucosal and myenteric ganglia of the small (Figure 7) and large intestine (Figure 8). LGR5 and LGR6 showed a bright cytoplasmic immunoreactivity predominantly in PGP9.5-labeled enteric neurons (Figure 7*Bi–Ci* and Figure 8*Bi–Ci*), yet less in S100beta-expressing glial cells (Figure 7*Bii–Cii*; Figure 8*Bii–Cii*). Furthermore, we detected LGR5 and LGR6 expression as fluorescent punctae in the neuropil (Figure 6*D and E*). In summary, LGR5/6 is particularly expressed in human enteric neurons, whereas LGR4 immunoreactivity was weak, but evenly distributed throughout enteric ganglia.

### FACS-purified LGR5 Negative ENS Cells Gave Rise to Newborn Neurons and Glial Cells

**Figure 9:**
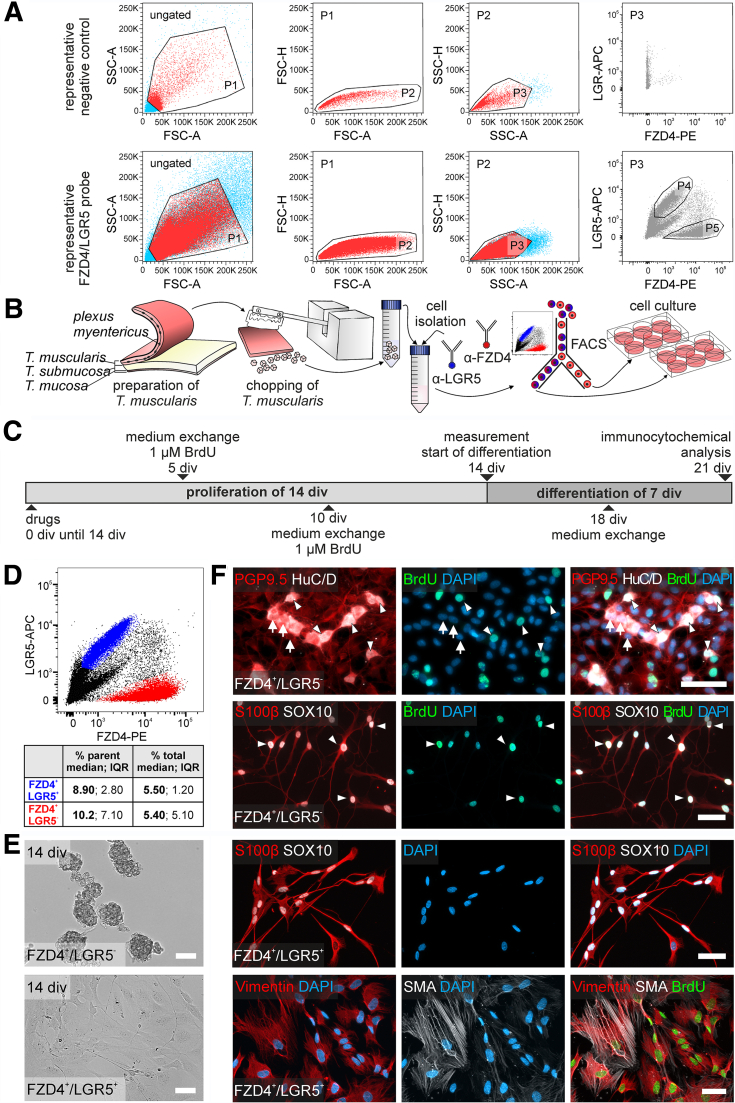
**FACS-purified LGR5^-^ human ENS cells give rise to newborn neurons and glial cells.** (*A–B*) Representative FACS of Fzd4 and LGR5 marked cells derived from human Tunica muscularis. The scatter blots represent the gating strategy (ungated, P1–P3) for Fzd4^+^LGR5^+^ (P4) and Fzd4^+^LGR5^-^ (P5) cell pools for the unstained control and the FZD4/LGR5-costained cell population. Each population that underwent further gating is colored in *red*. All events counted were first gated as P1 to exclude dead cells and debris, as these could be found at the bottom left corner of the first dot blot of each sample. Next, P1 was gated regarding its properties in forward (P2) and sideward (P3) scatter mode to exclude doublets and aggregates. Finally, P3 was gated regarding its FZD4/LGR5 property. (*B–C*) Sorted cell populations were proliferated until 14 div and differentiated for another 7 div. BrdU was added after 5 and 10 div. (*D*) Representative FACS-Blot of FZD4^+^LGR5^+^ (*blue*) vs FZD4^+^LGR5^-^ (*red*) cells. The cell populations were readily distinguishable and numerically stable across patients (Table in *B*; n = 5). (*E*) Representative brightfield images of FZD4^+^LGR5^+^ and FZD4^+^LGR5^-^ cell population after 14 div. FZD4^+^LGR5^-^ cultures gave rise to spheroids under proliferative conditions, whereas in FZD4^+^LGR5^+^ cultures, we found neural cells, arguably mature at isolation, and cells with smooth muscle/fibroblast-like morphology. **(***F*) Representative immunostaining for neuronal markers (PGP9.5, HuC/D, ß-III-tubulin), glial markers (S100beta, SOX10), fibroblasts and smooth muscle cells (vimentin, SMA), as well as BrdU and DAPI in FZD4^+^LGR5^+^ and FZD4^+^LGR5^-^ sorts after 21 div. We found BrdU-co-labeled Vimentin^+^ and SMA^+^ cells in the FZD4^+^LGR5^+^cell population, but not BrdU-incorporating neurons or glial cells, indicating that neurogenic and gliogenic cells were only found in the FZD4^+^LGR5^-^ population (*arrows*: BrdU^-^ neural cells; *arrowheads*: BrdU^+^ neurons). Scale bars: 50 μm. Source data are provided as a Source data file.

Considering the pro-proliferative RSPO1 effect on ENS progenitors, LGR-expressing cells might display their neuro/gliogenic potential only under a highly enriched in vitro microenvironment. Therefore, we established a FACS protocol for isolating LGR5-expressing cells from the human Tunica muscularis. We costained with the Wnt-receptor FRIZZLED4 (FZD4) as a useful marker to enrich enteric neural cells from Tunica muscularis of pediatric gut samples^38^ (Figure 9*A, B*). After FACS, purified cells proliferated for 14 div and differentiated for another 7 div. After 21 div, cell cultures were fixed and analysed (Figure 9*C*). We found that FZD4^+^LGR5^+^ cells (median, 8.90%; interquartile range [IQR], 2.80% of counts in parent population; n = 5) are a numerically stable cell pool clearly distinguishable from the FZD4^+^LGR5^-^ population (median, 10.2%; IQR, 7.10% of counts in parent population; n = 5) (Figure 9*D*). Under proliferative conditions, only the FZD4^+^LGR5^-^ cell population gave rise to free-floating spheroids (Figure 9*E*).

After differentiation, BrdU colabeling demonstrated that several cells were also TUJ1^+^, PGP9.5^+^, and/or HuC/D^+^, indicating that these cells divided and successively differentiated into neurons in vitro. However, most neurons were BrdU-negative. Moreover, we detected BrdU^+^SOX10^+^S100beta^+^ glial cells in FZD4^+^LGR5^-^-sorted cells (Figure 9*F*). In contrast, FZD4^+^LGR5^+^ cells were largely adherent under proliferative conditions, with a recognizable differentiated neuronal/glial morphology (Figure 9*E*). We were not able to detect any neurons in the FZD4^+^LGR5^+^ cell pool after differentiation, arguably due to a stressful FACS purification process. Yet, we found differentiated BrdU^-^ glial cells either SOX10^+^S100beta^+^ or only S100beta^+^. Moreover, these cultures were dominated by BrdU^+^SMA^+^Vimentin^+^ myofibroblast-like cells (Figure 9*E*). Thus, unlike the restricted LGR5 expression to proliferative epithelial ICS, LGR5 expression in the ENS was restricted to postmitotic cells. This raises the question if the observed RSPO1 effect is mediated by LGR4 or LGR6.

### LGR4- and LGR6-negative ENS Cells Were Neurogenic and Gliogenic in Culture

**Figure 10:**
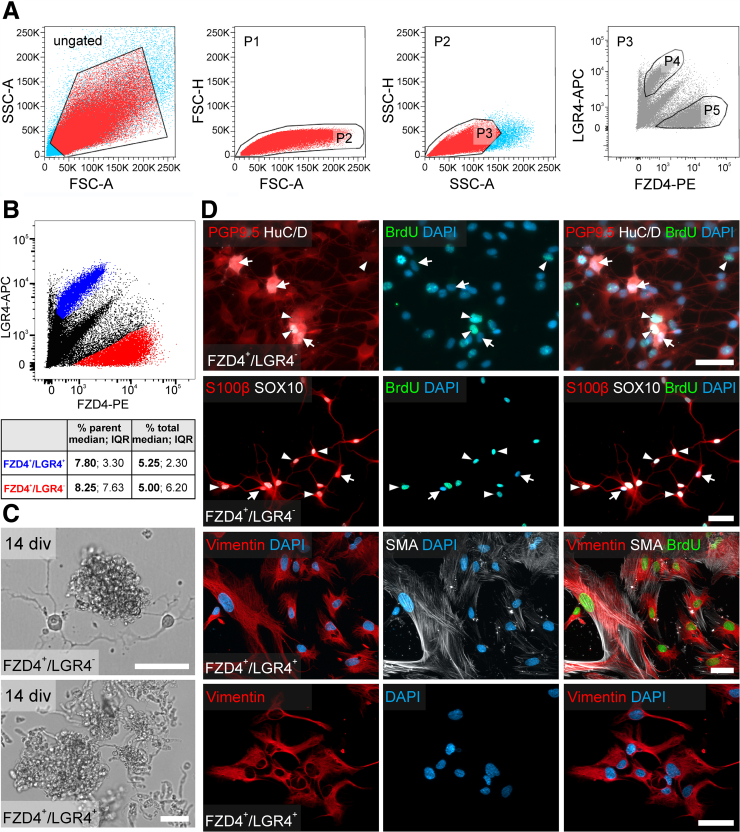
**Human LGR4^-^ ENS-cells are neurogenic and gliogenic.** (*A*) Representative FACS of Fzd4 and LGR4 marked cells derived from human Tunica muscularis. The scatter blots represent the gating strategy (ungated, P1–P3) for Fzd4^+^LGR4^+^ (P4) and Fzd4^+^LGR4^-^ (P5) cell pools. Each population that underwent further gating are colored in *red*. All events counted were first gated as P1 to exclude dead cells and debris, as these could be found at the bottom left corner of the first dot blot of each sample. Next, P1 was gated regarding its properties in forward (P2) and sideward (P3) scatter mode to exclude doublets and aggregates. Finally, P3 was gated regarding its FZD4/LGR6 property. (*B*) Representative FACS-Blot of FZD4^+^LGR4^+^ cells (*blue*), numerically stable across patients and readily distinguishable from FZD4^+^LGR4^-^ cells (*red*; Table in *A*; n = 4). (*C*) Only FZD4^+^LGR4^-^ cells formed spheroids cells after 14 div; FZD4^+^LGR4^+^ cultures largely exhibited cell debris. **(***D*) Micrographs depict representative immunostainings for neuronal markers (PGP9.5, HuC/D), glial markers (S100beta, SOX10), the fibroblast (vimentin) and smooth muscle cell marker (SMA), as well as BrdU and DAPI in FZD4/LGR4-sorted populations. Only FZD4^+^LGR4^-^ cells showed neurogenic and gliogenic potential (BrdU^+^, *arrowheads*); *arrows* indicate BrdU^-^ cells. FZD4^+^LGR4^+^-sorted cultures were dominated by BrdU^+^ and BrdU^-^ Vimentin^+^ and SMA^+^ cells. Scale bars: 50 μm. Source data are provided as a Source data file.

**Figure 11:**
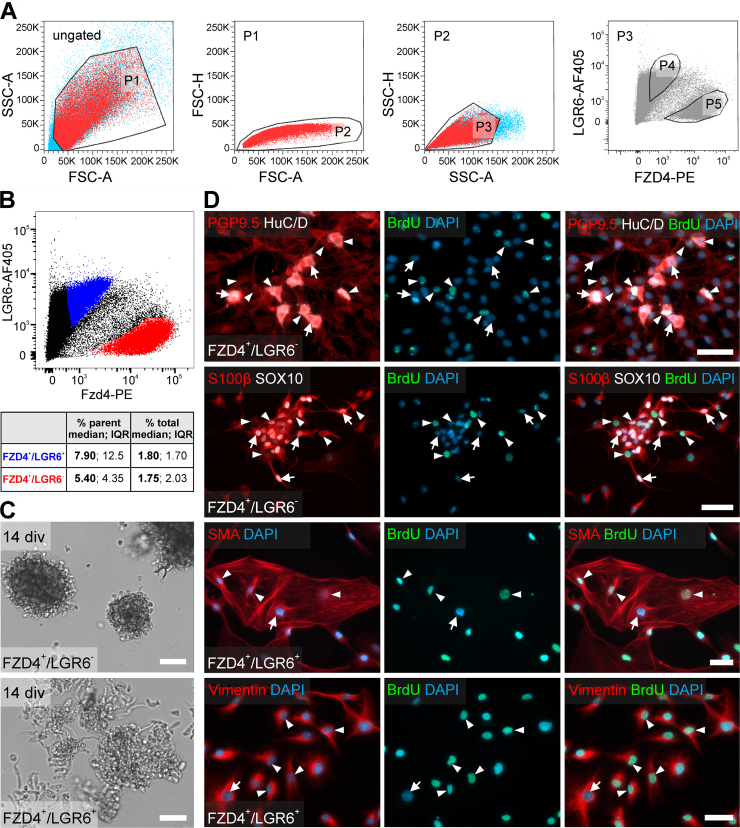
**Human LGR6^-^ ENS cells are neurogenic and gliogenic.** (*A*) Representative FACS of Fzd4 and LGR6 marked cells derived from human Tunica muscularis. The scatter blots represent the gating strategy (ungated, P1–P3) for Fzd4^+^LGR6^+^ (P4) and Fzd6^+^LGR6^-^ (P5) cell pools. Each population that underwent further gating is colored in *red*. All events counted were first gated as P1 to exclude dead cells and debris, as these could be found at the bottom left corner of the first dot blot of each sample. Next, P1 was gated regarding its properties in forward (P2) and sideward (P3) scatter mode to exclude doublets and aggregates. Finally, P3 was gated regarding its FZD4/LGR6 property. (*B*) Representative FACS blot of FZD4^+^LGR6^+^ cells (*blue*), numerically stable across patients and readily distinguishable from FZD4^+^LGR6^-^ cells (*red*; Table in *A*; n = 4). (*C*) Only FZD4^+^LGR6^-^ cells formed spheroids cells after 14 div; FZD4^+^LGR6^+^ cultures largely exhibited cell debris. (*D*) Micrographs depict representative immunostainings for neuronal markers (PGP9.5, HuC/D), glial markers (S100beta, SOX10), the fibroblast (vimentin) and smooth muscle cell marker (SMA), as well as BrdU and DAPI in FZD4/LGR6-sorted populations. Only FZD4^+^LGR6^-^ cells showed neurogenic and gliogenic potential (BrdU^+^, *arrowheads*); *arrows* indicate BrdU^-^ cells. FZD4^+^LGR6^+^-sorted cultures were dominated by BrdU^+^ (*arrowhead*) and BrdU^-^ (*arrow*) Vimentin^+^ and SMA^+^ cells. Scale bars: 50 μm. Source data are provided as a Source data file.

To validate LGR4 or LGR6 for their neuro/gliogenic potential, we FACS-purified FZD4^+^LGR4^+^ and FZD4^+^LGR6^+^ cells and cultured them as outlined above (Figure 10 and Figure 11). FZD4^+^LGR4^+^ cells and FZD4^+^LGR6^+^ cells were clearly distinguishable from the FZD4^+^LGR4^-^ cells (FZD4^+^LGR4^+^: median, 7.80%; IQR, 3.30% of counts in parent population, and FZD4^+^LGR4^-^: median, 8.25%; IQR, 7.63%; of counts in parent population n = 4) (Figure 10*A and B*) and the FZD4^+^LGR6^-^ cells (FZD4^+^LGR6^+^: median, 7.90%; IQR, 12.5% of counts in parent population, and FZD4^+^LGR6^-^ median: median, 5.40%; IQR, 4.35% of counts in parent population; n = 4) (Figure 11*A and B*). Again, only FZD4^+^LGR4^-^ and FZD4^+^LGR6^-^ cell population gave rise to spheroids during proliferation (Figures 10*C* and 11*C*). After differentiation, we detected BrdU^+^SOX10^+^S100beta^+^ (Figures 10*D* and 11*D*) and BrdU^-^S100beta^+^ glial cells in both LGR-negative populations. Additionally, we found BrdU^+^PGP9.5^+^HuC/D^+^ and BrdU^-^PGP9.5^+^HuC/D^+^ enteric neurons in the LGR-negative cultures (Figures 10*D* and 11*D*).

In contrast, cell sorting of FZD4^+^LGR4^+^ and FZD4^+^LGR6^+^ populations resulted mostly in nonviable cells during proliferation (Figures 10*C* and 11*C*). We were not able to detect neurons or glial cells in FZD4^+^LGR4^+^ and FZD4^+^LGR6^+^ sorted cell pool after differentiation. Again, these cultures were dominated by BrdU^+^Vimentin^+^ and BrdU^+^SMA^+^Vimentin^+^ myofibroblast-like cells (Figures 10*D* and 11*D*).

Thus, our FACS data combined with our immunohistochemical analysis showed that especially LGR5 and LGR6 receptors are present on differentiated neurons in vivo. Moreover, LGR4 is weakly expressed in the postnatal ENS in vivo. To explain the apparent contradiction between our in vivo findings vs the RSPO1-mediated pro-proliferative effect in vitro, we conducted the following experiments to test if this might be caused by a culture-dependent LGR receptor expression on ENS cells during proliferation and differentiation.

### LGR4 and LGR5, but not LGR6, Are Expressed in Proliferative ENS Progenitors

**Figure 12:**
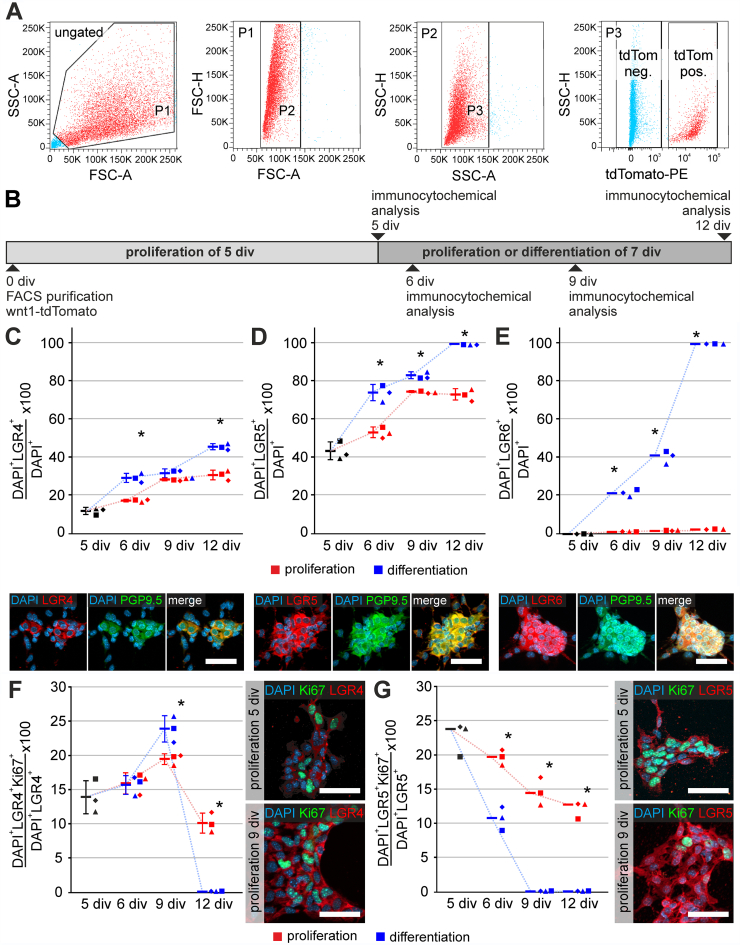
**Different roles of LGR4, LGR5, and LGR6 in ENS progenitor proliferation and differentiation.** (*A*) Representative FACS of tdTomato expressing neural ENS cells derived from postnatal day 0 old Wnt1Cre2 mice. The scatter blots represent the gating strategy (ungated, P1–P3) for tdTomato negative (P4) and tdTomato positive (P5) cell pools. Each population that underwent further gating is colored in *red*. All events counted were first gated as P1 to exclude dead cells and debris, as these could be found at the bottom left corner of the first dot blot of each sample. Next, P1 was gated regarding its properties in forward (P2) and sideward (P3) scatter mode to exclude doublets and aggregates. Finally, P3 was gated regarding its tdTomato-expressing property. See also the supplementary data file that summarizes the gating data for all experiments used in this study. (*B*) Murine ENS neurospheres (P0, wnt1-tomato) were adherently proliferated for 5 div. Afterwards, one group was kept under proliferation conditions until 6 div, 9 div, and 12 div, whereas the second group was differentiated. (*C–E*) Percentages of LGR4^+^ (*C*), LGR5^+^ (*D*), and LGR6^+^ (*E*) cells of all DAPI^+^ cells throughout proliferation (*red*) and differentiation (*blue*). Asterisk indicates significance (proliferation vs differentiation); bars and error bars are mean ± SD. Independent replicates are represented by different symbols. Micrographs depict representative LGR staining, PGP9.5, and DAPI after 9 div during differentiation. The percentages of LGR4^+^/DAPI^+^ and LGR5^+^/DAPI^+^ cells increased during proliferation and differentiation (ANOVA, Fisher LSD; bars and error bars are mean ± SD; n = 3). LGR6 expression was absent during proliferation, but increased during differentiation (ANOVA on Ranks, Student-Newman-Keuls; bars are medians; n = 3). (*F–G*) Dot plots illustrate the percentages of proliferative, Ki67^+^/LGR4^+^ (*F*) or Ki67^+^/LGR5^+^ (*G*) cells among LGR4^+^ or LGR5^+^ cells, throughout proliferation (*red*) and differentiation (*blue*). Asterisk indicates significance (proliferation vs differentiation); bars and error bars are mean ± SD. Independent biological replicates are represented by different symbols. Micrographs depict representative LGR staining, Ki67, and DAPI on 5 div and 9 div during proliferation. The percentage of Ki67^+^/LGR4^+^ cells increased during proliferation and differentiation until 9 div (ANOVA, Fisher LSD, bars and error bars are mean ± SD; n = 3). In contrast, the percentage of Ki67^+^/LGR5^+^ cells decreased constantly in vitro (ANOVA on Ranks, Student-Newman-Keuls, bars indicate medians; n = 3). Source data are provided as a Source data file.

To assess the regulation of LGR receptors*,* we used FACS-purified tdTomato^+^ ENS cells from wnt1-tomato reporter mice (P0; Figure 12*A*), seeded them on collagen-coated cover slips, and proliferated them for 5 div. Afterwards, one group was kept under proliferative conditions, whereas the other group entered differentiation. After 6 div, 9 div, and 12 div, cultures were fixed, and LGR4/5/6-receptor expression was evaluated by immunocytochemistry (Figure 12*B*).

LGR4-receptor was expressed only on very few cells after 5 div; nevertheless, its expression increased throughout the culture, irrespective of the culture setup. At the end of differentiation, we detected LGR4 expression in differentiated PGP9.5-positive neurons (ANOVA, Fisher LSD post-hoc test, mean ± SD proliferation vs differentiation: 5 div: 11.5% ± 1.85%; 6 div: 17.0% ± 0.75% vs 28.8% ± 2.40% [*P* ≤ .001]; 9 div: 28.0% ± 0.68% vs 31.3% ± 2.25% [*P* = .047]; 12 div: 30.4% ± 2.56% vs 45.2% ± 1.60% [*P* ≤ .001]; n = 3) (Figure 12*C*). Interestingly, LGR5 receptor expression showed a similar trajectory as LGR4. However, LGR5 was more prominent after 5 div. Further, at the end of differentiation (12 div), nearly every PGP9.5^+^ neuron expressed LGR5 (ANOVA, Fisher LSD post-hoc test, mean ± SD proliferation vs differentiation: 5 div: 44.1% ± 4.67%; 6 div: 53.8% ± 2.75% vs 74.5% ± 4.25% [*P* ≤ .001]; 9 div: 75.1% ± 0.49% vs 83.6% ± 1.78% [*P* = .003]; 12 div: 73.6% ± 3.01% vs 100% ± 0.00% [*P* ≤ .001]; n = 3) (Figure 12*D*).

In contrast to LGR4 and LGR5, LGR6 expression was hardly present during proliferation; however, its expression increased during differentiation until almost every cell expressed LGR6 after 12 div (ANOVA on Ranks, post-hoc: Student-Newman-Keuls Method, median proliferation vs differentiation: 5 div: 0.01%; 6 div: 1.32% vs 21.7% [*P* ≤ .05]; 9 div: 19.1% vs 41.4% [*P* ≤ .05]; 12 div: 25.6% vs 100% [*P* ≤ .05]; n = 3) (Figure 12*E*). To evaluate proliferation in these cultures, we analysed the expression of Ki67 in LGR4/5/6-expressing cells (Figure 12*F*). The percentage of Ki67^+^LGR4^+^ cells increased steadily until 9 div under proliferative and differentiation conditions. By 12 div, proliferation rapidly decreased, possibly due to contact inhibition (ANOVA, Fisher LSD post-hoc test, mean ± SD proliferation vs differentiation: 5 div: 13.9% ± 2.41%; 6 div: 15.9% ± 1.54% vs 15.6% ± 1.37% [*P* = 0.853]; 9 div: 19.4% ± 0.78% vs 23.8% ± 1.91% [*P* = 0.003]; 12 div: 10.1% ± 1.44% vs 0.12% ± 0.01% [*P* ≤ .001]; n = 3) (Figure 12*F*).

In contrast, the percentage of Ki67^+^LGR5^+^ cells constantly decreased in culture, and we detected hardly any Ki67^+^LGR5^+^ cell after 9 div in differentiation conditions (ANOVA on Ranks, post-hoc: Student-Newman-Keuls Method, median proliferation vs differentiation: 5 div: 23.8%; 6 div: 19.7% vs 10.8% [*P* ≤ .05]; 9 div: 14.4% vs 0.02% [*P* ≤ .05]; 12 div: 12.7% vs 0.05% [*P* ≤ .05]; n = 3) (Figure 12*G*). We did not detect proliferative LGR6-positive cells.

### LGR4^+^ and Less so LGR5^+^ Cells Are Drivers of RSPO1-induced Neural Proliferation

**Figure 13:**
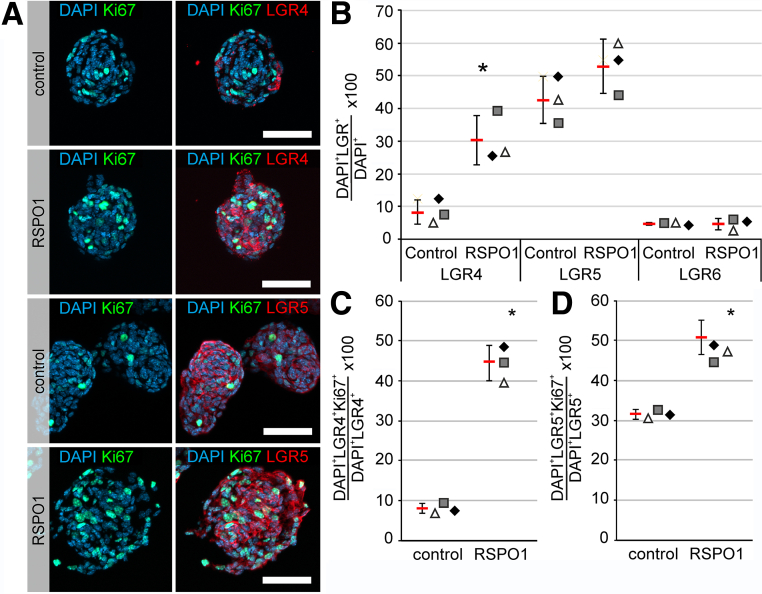
**RSPO1 stimulation increases LGR4 and LGR5 receptor expression in murine ENS cells in vitro.** (*A*) RSPO1-treated and unstimulated enterospheres were cultivated under proliferative conditions for 5 div. Fixed-frozen cryosections of these enterospheres were stained for LGR4 (*red*), LGR5 (*red*), LGR6, and the nuclear marker DAPI (*blue*). (*B*) The bar graph represents the quantification of LGR4^+^, LGR5^+^, and LGR6^+^ cells under control and RSPO1-treated condition. Asterisk indicates significant difference of LGR-expression in RSPO1-stimulated enterospheres compared with control. Data points for independent biological replicates are represented by different symbols. RSPO1 stimulation enhanced significantly the percentage of LGR4^+^-expressing cells (ANOVA, Fisher LSD post-hoc test, mean ± SD; n = 3). The number of LGR5^+^ cells increased, but not statistically significant (ANOVA, Fisher LSD post-hoc test, mean ± SD; n = 3). However, the number of LGR6-expressing cells was not affected by RSPO1 stimulation, arguably due to the small effect size of 3 independent experiments (LGR6: ANOVA, Fisher LSD post-hoc test, mean ± SD; n = 3). (*C–D*) RSPO1-stimulated and unstimulated enterospheres were cultured for 5 div. Fixed-frozen cryosections were stained for LGR4 or LGR5 (*red*), Ki67 (*green*), and DAPI (*blue*). Asterisk indicates significant difference to control. Data points for independent replicates are represented by different symbols. RSPO1 stimulation enhanced the percentage Ki67^+^LGR4^+^ by 5.40-fold and Ki67^+^LGR5^+^ cells by 1.62-fold (ANOVA, Fisher LSD, bars and error bars are mean ± SD; n = 3). Scale: 50 μm. Source data are provided as a Source data file.

Finally, to assess whether proliferative LGR4- or LGR5-expressing cells are responsive to RSPO1 stimulation, we used fixed-frozen cryosections of enterospheres proliferated for 5 div and stimulated with RSPO1. Untreated enterospheres served as controls. Immunocytochemical analysis for LGR4 and LGR5 receptor (Figure 13*A*) showed that the percentage of LGR4^+^DAPI^+^ and LGR5^+^DAPI^+^ cells increased in RSPO1-stimulated spheres compared with controls (LGR4^+^DAPI^+^: ANOVA, Fisher LSD post-hoc test, mean ± SD: Control: 8.19% ± 3.72%; RSPO1: 30.3% ± 7.53%; n = 3; [*P* = .010]; LGR5^+^DAPI^+^: ANOVA, Fisher LSD post-hoc test, mean ± SD: Control: 42.6% ± 7.02%; RSPO1: 52.8% ± 8.24%; n = 3; [*P* = .179]) (Figure 13*B*). The percentage of rarely found LGR6^+^DAPI^+^ cells did not change (LGR6: ANOVA, Fisher LSD post-hoc test, mean ± SD: Control: 4.64% ± 0.32%; RSPO1: 4.53% ± 1.74%; n = 3; [*P* = .918]) (Figure 13*B*). Surprisingly, RSPO1 stimulation impressively increased the percentage of Ki67^+^LGR4^+^ by 5.40-fold (ANOVA, Fisher LSD post-hoc test, mean ± SD: Control: 8.26% ± 1.31%; RSPO1: 44.6% ± 4.37%; n = 3; [*P* ≤ .001]) (Figure 13*C*), whereas it was far less drastic in Ki67^+^LGR5^+^ cells (1.62-fold; ANOVA, Fisher LSD post-hoc test, mean ± SD: Control: 31.3% ± 1.20%; RSPO1: 50.6% ± 4.80%; n = 3; [*P* = .002]) (Figure 13*D*), indicating that the pro-proliferative effect of RSPO1 is primarily mediated by LGR4-expressing cells. Therefore, our results indicate that RSPO1 acts on ENS homeostasis depending on the LGR receptor expressed: while LGR5 and LGR6 might play a role in neuronal differentiation and maintenance, LGR4 arguably mediates pro-proliferative signals and is itself upregulated in proliferative/regenerative microenvironments.

## Discussion

Recent spatial mRNA profiling revealed that WNT signaling components, including RSPOs and LGRs, are expressed in the postnatal ENS,^32^ suggesting a role of RSPO-LGR signaling in ENS homeostasis. However, LGR receptor knockouts are associated with severe developmental deficiencies also in the gastrointestinal tract^39^^,^^40^ and are not viable until the desired postnatal age. Hence, isolated postnatal murine and patient-derived ENS progenitors are highly valuable, to evaluate cellular and molecular characteristics of RSPO1-LGR signaling. Previously we described the expression of Wnt and RSPO signaling components in postnatal ENS progenitors in vitro,^31^^,^^37^ which complements our current results on the presence of *Rspo* ligands and *Lgr4/5/6* receptors in enterospheres, underlining the translational value of this culture model.

Furthermore, we detected the *Znrf3* ligase mRNA expression in our enterosphere model. The transmembrane ligase ZNRF3 has been reported to shut down WNT signaling activity.^34^ In this context, Hao et al reported a stabilizing function of RSPO1-LGR4 interaction on FZD receptors by blocking *Znrf3* ligase activity. In their in vitro model, RSPO1-LGR4 interaction enhanced canonical and noncanonical Wnt signaling activity via increased expression especially of FZD4, as well as FZD6 and FZD8 receptors.^34^ In our study, we did not assess downstream signaling mediators to validate the general effect of RSPO1 on canonical or noncanonical WNT signaling activity. Yet, considering the reported positive effect, especially on FZD4 receptor expression, highlights future experiments that should focus on downstream signaling mediators of the RSPO1-LGR4-FZD4-ZNRF3 axis to unravel in detail canonical and noncanonical WNT signaling interaction partners.

### Cellular Effect of RSPO1 Stimulation on Murine and Human ENS Cells

Here, we showed that RSPO1 has a pro-proliferative effect on postnatal murine ENS progenitors, leading to a higher yield of newborn BrdU^+^HuC/D^+^ neurons in vitro. In addition, we found that the pro-proliferative and neurogenic effect of RSPO1 on ENS cells derived from newborn and adult mice barely differ. Thus, our data highlights a quite robust neurogenic ENS cell population that could be recruited by RSPO1 stimulation and thus might be activatable in a regenerative setting throughout postnatal life, at least within the observed age frame.

It is noteworthy that, although the pro-proliferative effect of RSPO1 on P75-expressing neural cells was readily detectable and statistically significant using the proliferation marker Ki67, the increase in the total P75-positive population compared with all DAPI-positive cells was modest and did not reach statistical significance. This is likely due to the early timepoint of our measurement, at which a statistically higher proliferative rate did not yet translate to statistically detectable increases in the total cell population. It is conceivable that longer culturing times would increase the contrast between the groups. Moreover, within the Tunica muscularis, P75 expression can be found in neural cells, particularly in progenitor and/or glial cells,^41^ whereas it is downregulated during neuronal differentiation and remains on a low expression level in enteric neurons.^42^ Thus, it is conceivable that RSPO1 is activating progenitor and/or glial proliferation, but also neuronal differentiation. Thereby, the increase in the total number of P75-positive cells after RSPO1 stimulation might be lessened by a subpopulation of late progenitor cells losing detectable P75 expression as they differentiate into mature neurons. Nevertheless, our results on neuronal cell count and neurogenesis underline that even short RSPO1 stimulation periods are sufficient to induce clearly detectable neurogenesis in ENS cells.

Moreover, the pro-proliferative and neurogenic effect of RSPO1 was reproducible in human ENS progenitors, thereby suggesting a conserved mechanism within mammals. Interestingly, we detected an additive effect of RSPO1 and WNT3A in the human as well as the neonate murine enterosphere model, whereas the combined treatment in the adult murine model led to less differentiated neurons compared with the either of the WNT agonists alone. Because the neonate murine and the human cells were both isolated during the early postnatal phase, it is conceivable that a strong activation or overactivity of the WNT pathway is beneficial for neurogenesis, whereas in older/mature animals the same level of WNT activation would trigger a rapid downregulation, which interferes with neuronal differentiation, or might even become toxic. Indeed, excess WNT signaling was found to trigger cell cycle re-entry in differentiating neural progenitors in the CNS and may contribute to neurological diseases.^43^, ^44^, ^45^ Likewise, it is possible that restraining control mechanisms counteract uncontrolled proliferation in a mature ENS.

Interestingly, similar pro-proliferative RSPO1 effects have been reported in neural stem cells (NSCs) from the adult murine hippocampus, where proliferative NSCs expressed LGR4/5 receptors, while neighboring astrocytes shaped a pro-proliferative microenvironment by secreting RSPO1.^46^ Thus, an ENS progenitor niche with secreted Wnt and RSPO ligands, as well as respective receptors on neural progenitors, is conceivable. Indeed, Stavely et al reported that EMCs secrete Wnt ligands, *Rspo1* and *Rspo3*, whereas *Frizzled* and *Lgr* receptors are expressed on ENS progenitors.^33^ Additionally, our results on FACS-purified murine ENS neurospheres suggest that ENS cells themselves secret Wnt ligands. This is supported by our recently published mRNA expression atlas showing that Wntless *(Wls),* which is indispensable for transportation and secretion of Wnt-ligands, is expressed in murine enteric ganglia.^32^ Interestingly, this mRNA expression study showed that *Lgr5* and *Lgr6* transcripts displayed a distinct localization within enteric neuronal perikaryal,^32^ which is in line with the immune stainings on human gut sections in the current study. Moreover, single-cell RNA-sequencing data demonstrated the expression of *Rspos* and *Lgr*-receptors in enteric neuronal and glial cell clusters.^47^ Therefore, enteric neurons and glial cells might be source and receiver of RSPO signaling in the ENS.

### Role of LGR4/5/6 Receptor Expression During Postnatal ENS Maturation

Differentiation of NSCs in the murine hippocampus is associated with downregulation of LGR4/5 receptor expression, contributing to the attenuation of the canonical Wnt/β-catenin signaling pathway observed in late neural progenitors.^48^^,^^49^ Similar results have been described for the LGR5-dependent maturation of adult epithelial ISCs,^50^ whereby RSPO and WNT ligands have qualitatively distinct, noninterchangeable roles. Although WNT proteins maintain LGR5 receptor expression on ISCs, RSPO ligands augment ongoing WNT signaling, thereby actively controlling the extent of stem cell expansion.^51^ Moreover, RSPO inhibition induces lineage commitment and differentiation by reduced WNT signaling activity.^52^

Previously, we showed that canonical WNT signaling is inactivated in enterospheres after the induction of cellular differentiation.^30^ Strikingly, in the current study, the observed pro-proliferative effect of RSPO1 was paralleled by a reduced rate of actively cycling ENS cells expressing LGR5 in vitro. However, we observed that LGR5 was increasingly expressed over the course of enteric neuronal differentiation, which correlates with the observed in vivo expression in human gut specimens. Thus, LGR5-expressing ENS cells might be either less proliferative, or are in a late proliferation phase, ready to leave the cell cycle. Still, these comprise a small proportion of cells recruitable to a proliferative state by RSPO1 stimulation. Interestingly, noncycling ISCs can also express LGR5. These cells are quiescent precursors that committed to the Paneth cell and enteroendocrine lineages. Notably they can be recalled to the stem cell state after injury.^50^ Intriguingly, in the central nervous system, LGR5 expression is predominantly found in postmitotic neurons, partly supporting our results. Miller and colleagues reported that LGR5 and RSPO1 are expressed in murine cerebellar granule neurons. Of note, this expression was restricted to the Wnt-dependent maturation phase and was not observed in adults.^53^ Furthermore, predominantly LGR5 expression has been reported in the neurogenic area of the olfactory bulb. However, LGR5 labeling was not detected in stem cells but in fully differentiated neurons of various subtypes and maturation stages.^54^ In contrast to several studies suggesting LGR6-receptor as a stem cell marker and tumor suppressor,^55^^,^^56^ we barely found LGR6 expression in ENS cells during proliferation in vitro. Similar results have been described for murine adult hippocampal NSCs, where LGR6 exhibit the lowest expression levels of LGR4/5/6 during proliferation in vitro.^46^ Moreover, we detected that LGR6 expression increased during differentiation suggesting a yet to be elucidated function in neuronal maturation.

Surprisingly, we detected LGR4 receptor expression in actively cycling ENS cells, which increased during proliferation in vitro. Further, although its expression also enhanced towards differentiation of ENS cells, it was hardly detectable in the mature ENS in vivo. Interestingly, murine knockout studies for LGR4 or LGR5 receptor have shown that LGR4 and LGR5 receptors fulfil comparable functions at least during fetal and postnatal development of the ISCs niche and could compensate each other.

However, LGR4 deficiency could not be rescued by LGR5 function in the adult mouse^24^^,^^57^; hence, highlighting different LGR4/5-receptor functions during proliferation, cell cycle exit, and fate commitment. LGR4-mediated WNT signaling has been reported to function as a protective factor in tissue homeostasis of the gastrointestinal tract in vivo.^58^

LGR4-deficient mouse models were prone to dextran sodium sulfate (DSS)-induced inflammatory bowel disease (IBD),^58^ related to impaired proliferation and differentiation of intestinal crypts and Paneth cells during tissue regeneration. RSPO1 administration in these mouse models accelerated mucosal regeneration and restoration of intestinal architecture, arguably by restored WNT signaling activity and stem cell function.^59^ To what extent LGR receptors alone or cooperatively execute proliferation-dependent functions in ENS progenitors is not assessable with the presented study. Altogether, our results indicate that RSPO1-mediated signaling might have different functional outcomes during ENS progenitor proliferation and fate commitment via LGR4/5, as well as in maintenance of differentiated ENS cells arguably via LGR6 expression.

### Clinical Implications

Our study showed that the pro-proliferative effect of RSPO1 in vitro might be mediated via LGR4/5 receptor. However, LGR4 was hardly expressed in vivo, and LGR5/6 receptor expression was particularly found in mature enteric neurons. Additionally, isolated and FACS-sorted LGR-expressing human ENS cells did not exhibit a proliferative and neurogenic behavior. This apparent contradiction between in vivo vs in vitro results suggests that the developmental and/or regenerative influences of the surrounding cellular microenvironment defines LGR receptor expression. Thereby, the pro-proliferative in vitro environment triggers LGR expression in ENS cells, which in turn exert their proliferative and neurogenic function. This notion is supported by our experiments showing that LGR4 and LGR5 are markedly upregulated in proliferative culture conditions, whereas LGR6 becomes detectable during induced differentiation only. Thus, unraveling the defining molecular markers that hallmark a homeostatic versus a regenerative/neurogenic micro milieu will be essential for future therapeutic approaches.

Although we did not focus on the role of RSPO-LGR signaling on enteric glial cells, the in vivo expression data in this study, as well as recently published data,^19^^,^^32^ highly suggest a crosstalk with enteric glial cells. Baghdadi et al highlighted the expression of several Wnt ligands by enteric glial cells in the Lamina propria mucosae. This in turn maintains the self-renewal capacity of epithelial ISCs and influences epithelial regeneration and barrier function in mice.^19^ Whether enteric glial cells also communicate bidirectionally with enteric neurons via the WNT signaling pathway to influence neuronal or glial homeostasis during postnatal maturation remains elusive. In this context, future stimulation experiments with RSPO1 on separately sorted glia and neuron populations are needed to further distinguish the role of each cell population in our in vitro model.

Yet, enteric glial communication with long-lived anti-inflammatory M2 macrophages in the Tunica muscularis has been recently outlined to control maintenance of ENS integrity, structure, and function.^3^ Of note, RSPO1-LGR4 receptor signaling has been reported to initiate a phenotype switch of anti-inflammatory M2 macrophages due to tissue inflammation.^60^ Furthermore, alterations in this phenotype have been implicated in gastrointestinal motility disorders like postoperative ileus (POI).^61^ Interestingly, Leven and Schneider et al demonstrated that enteric glial cells upregulate *LGR4* and Ki67 expression during the inflammatory manifestation of enteric gliosis in a model of POI. Thereby, the amount of proliferative Ki67^+^/SOX10^+^ cells increased 24 hours after POI induction, which was further reflected in an increase in glial cell number during the recovery timepoint 72 hours later compared with 24 hours.^62^ Thus, a link between RSPO-LGR signaling to intestinal inflammation and neural proliferation is conceivable, although the underlying mechanism is yet to be unravelled. Nevertheless, our data suggests that LGR4 expression could be an indicator for an imminent cell division, arguably triggered by highly proliferative/regenerative conditions (eg, inflammatory microenvironments or cell cultures). To clarify the in vivo role of LGR4/5/6 in ENS homeostasis, conditional knockouts of single LGRs and LGR combinations are needed in future studies. Using ENS-specific, inducible knockout models would thereby help to circumvent early foetal lethality or perturbations caused by conventional LGR knockouts and would also allow for the targeted deletion of LGR expression at designated time points of regeneration/proliferation or neuronal differentiation.

Taken together, our results highlight that LGR receptors serve a diverse function in cellular maturation of postnatal ENS progenitors in vitro. Thereby differential LGR receptor expression could function as indicators for a turning point in ENS progenitor fate: a fork stuck in the road either to proliferation or neuronal differentiation.

## Materials and Methods

### Animals

Animals were handled in accordance with national and international guidelines (Notification number AT 01/19 M). Mice were housed in standard cages with standard pathogen-free breeding and a standard 12-hour light/dark cycle at 22 ± 2°C and 60% ± 5% humidity. Germ-free food and water were available ad libitum. For isolation of unpurified ENS cultures neonatal (P0-P5, postnatal day 0-5) C57BL/6J mice were used without regard to sex. For FACS-based isolation of ENS cells, we crossbred B6;129S6-Gt(ROSA)26Sortm9(CAG-tdTomato)Hze/J (Jackson Laboratory; stock no. 007914) mice with B6.Cg-Tg(Wnt1-cre)2Sor/J (Jackson Laboratory; stock no. 022501) mice. The F1 offsprings expressed the red-fluorescent protein tdTomato within all ENS cells and are termed *wnt1-tomato* in this work. For isolation of purified ENS cultures, neonatal (P0-P5, postnatal day 0-5) and 2-month-old (P60, postnatal day 60) wnt1-tomato mice were used without regard to sex.

### Human Specimens

Human gut samples were obtained from 16 male and 14 female patients aged between 3 days and 12 years, who were operated due to imperforate anus, intestinal obstruction syndrome, or short-gut syndrome (Table 2). All samples were collected after approval by the local ethical committee (Project Nr. 652/2019BO2 and 066/2023BO2), and according to the declaration of Helsinki.

**Table 2:** Intestinal Resectates Used in This Study

Age	Sex	Diagnosis	Gut region	Experiment
3 days	Female	Imperforate anus	Transverse colon	Cell culture
9 months	Male	Obstruction syndrome	Transverse colon	Cell culture
5 months	Male	Imperforate anus	Transverse colon	Cell culture
5 months	Female	Imperforate anus	Transverse colon	Cell culture
3 years / 11 months	Female	Obstruction syndrome	Transverse colon	Fzd4-LGR4 (n1)
12 years / 6 months	Female	Familial adenomatous polyposis	Ileum	Fzd4-LGR4 (n2)
12 years / 3 months	Male	Obstruction syndrome	Transverse colon	Fzd4-LGR4 (n3)
4 months	Male	Obstruction syndrome	Ileum	Fzd4-LGR4 (n4)
2 years / 4 months	Female	Imperforate anus	Ileum	Fzd4-LGR5 (n1)
1 year	Male	Imperforate anus	Ileum	Fzd4-LGR5 (n2)
9 months	Female	Obstruction syndrome	Transverse colon	Fzd4-LGR5 (n3)
1 year / 1 month	Male	Obstruction syndrome	Descendent colon	Fzd4-LGR5 (n4)
2 years / 9 months	Male	Obstruction syndrome Hirschsprung condition	Ileum	Fzd4-LGR5 (n5)
16 years / 4 months	Male	Obstruction syndrome	Ileum	Fzd4-LGR6 (n1)
3 months	Male	Imperforate anus	Ileum	Fzd4-LGR6 (n2)
11 months	Male	Imperforate anus	Transverse colon	Fzd4-LGR6 (n3)
2 years / 1 month	Male	Obstruction syndrome Hirschsprung condition	Ileum	Fzd4-LGR6 (n4)
1 year	Female	Imperforate anus	Transverse colon	Histology
3 months	Female	Imperforate anus	Duodenum	Histology
1 year / 2 months	Female	Rhabdomyosarcoma	Transverse colon	Histology
2 months	Male	Imperforate anus	Descendent colon	Histology
6 months	Male	Obstruction syndrome	Transverse colon	Histology
1 year / 6 months	Female	Obstruction syndrome	Jejunum	Histology
4 months	Male	Imperforate anus	Transverse colon	Histology
7 months	Female	Imperforate anus	Transverse colon	Histology
3 years / 8 months	Male	Obstruction syndrome	Jejunum	Histology
9 months	Female	Imperforate anus	Transverse colon	Histology
4 months	Female	Imperforate anus	Ileum	Histology
11 months	Female	Obstruction syndrome	Ileum	Histology
5 months	Male	Imperforate anus	Descendent colon	Histology

### Cell Culture of Enteric Neural Progenitors

ENS progenitors from mice and human patients were isolated from the Tunica muscularis and cultured as previously described^37^^,^^38^ (see detailed description in the following paragraphs). The culture of unpurified murine and human Tunica muscularis cells (including ENS progenitors) under proliferation conditions resulted in 3-dimensional spheroids, termed enterospheres. In contrast, 3-dimensional spheroids, derived from FACS-purified neural crest-derived cells were termed neurospheres. Enterosphere-cultures also comprised non-neuronal cells of the Tunica muscularis such as smooth muscle cells or fibroblasts (but not submucosal and mucosal cells, such as epithelial cells). This is an advantage, as it resembles the environment of the in vivo situation more accurately. Based on age and species used for in vitro experiments, enterospheres and neurospheres are subjected to different proliferation periods to generate a robust amount of proliferating ENS cells for subsequent analysis.

### Cell Isolation of Murine Enteric Neural Progenitors

Postnatal day 0- to 5-day-old mice were killed by decapitation. P60 mice were anesthetized with isoflurane, followed by cervical dislocation. The whole intestine convolute was removed and transferred in murine preparation buffer (Hanks’ balanced salt solution [HBSS] without Ca^2+^/Mg^2+^ [Sigma-Aldrich], penicillin [100 U/mL; PAA], streptomycin [100 mg/mL; Sigma-Aldrich]). Adherent mesenteria were dissected, and the longitudinal and circular muscle layers containing myenteric plexus were stripped off the small intestine and collected in murine preperation buffer. After chopping, tissue was enzymatically digested in collagenase type XI (750 U/mL; Sigma-Aldrich)/dispase type II (250 mg/mL; Roche Diagnostics) solved in HBSS with Ca^2+^/Mg^2+^ (Sigma-Aldrich) for 20 minutes at 37°C. Tissue was carefully triturated every 10 minutes with a fire-polished 1-mL pipette tip. Before the first trituration step, cell suspension was treated with 0.05% (w/v) DNase I (Sigma-Aldrich). After tissue dissociation, 10% (v/v) fetal calf serum ([FCS], Biochrom) was added. To remove any residual enzymes, 2 washing steps in HBSS without Ca^2+^/Mg^2+^ were performed at 200 g. Finally, undigested larger tissue pieces were removed with a 30-μm cell strainer (Miltenyi Biotec GmbH). The pellet was resuspended in murine proliferation media (Dulbecco’s modified Eagle’s medium with Ham’s F12 medium [DMEM] 1:1; Life technologies) containing N2 supplement (1:100; Life Technologies), penicillin (100 U/mL), streptomycin (100 mg/mL), L-glutamine (2 mM; Sigma-Aldrich), EGF (20 ng/mL; Sigma-Aldrich), and hbFGF (20 ng/mL; Sigma-Aldrich). Cells were seeded at a concentration of 2.0 × 10^4^ cells/cm^2^, and the media was supplemented with B27 (1:50; Gibco Thermo Fisher Scientific) once before seeding. Cells were cultured up to 5 div under proliferation conditions, whereby growth factors (20 ng/mL hEGF/20 ng/mL hbFGF) were added daily. 1 μM BrdU was added after 2 div during the proliferation phase.

### Cell Isolation of Human Enteric Neural Progenitor Cells

For human specimen, the resectates were cut open along the longitudinal axis and rinsed twice with human preparation buffer (HBSS without Ca^2+^/Mg^2+^, penicillin (100 U/mL), streptomycin (100 mg/mL), cibrofloxacin Kabi (5 mm/mL; Fresenius Kabi), and metronidazole (50 mg/mL; B. Braun). The Tunica adventitia and scar tissue were removed, and the Tunica muscularis was peeled off the Tela submucosa. Tunica muscularis preparations were then stored in human preparation medium at 4°C overnight. On the next day, the Tunica muscularis was chopped multiple times (800 mm each) using a Mcllwain tissue chopper (Mickle Laboratory Engineering Co). The pieces were enzymatically digested in collagenase type XI (750 U/mL)/dispase type II (250 mg/mL) dissolved in HBSS with Ca^2+^/Mg^2+^ containing 0.05% (w/v) DNase I and incubated for up to 60 minutes at 37°C. Tissue was triturated every 20 minutes with a fire-polished 25-mL serologic pipette.

After tissue dissociation, FCS was added to a concentration of 10% (v/v). The cells were pelleted at 200 g, and erythrocyte lysis was performed using RBC Lysis buffer (eBioscience). After a second centrifugation at 200 g, the pellet was resuspended in HBSS without Ca^2+^/Mg^2+^ and filtered using 100-μm and 70-μm cell strainers. Cells were pelleted again at 200 g and resuspended in human proliferation medium (DMEM containing N2 supplement [1:100], penicillin [100 U/mL], streptomycin [100 mg/mL], L-glutamine [2 mM], EGF [20 ng/mL], and hbFGF [20 ng/mL]). Cell suspension was finally filtered using a 30-μm cell strainer and seeded in a concentration of 2.0 × 10^4^ cells/cm^2^. The medium was supplemented with B27 (1:50) once before seeding. Cells were cultured up to 14 div under proliferation conditions, whereby growth factors (20 ng/mL hEGF/20 ng/mL hbFGF) were added daily, and culture medium was exchanged every 5 days. 1 μM BrdU was added after 5 div and 10 div during the proliferation phase.

### FACS of Purified Murine Cultures

For FACS analysis, cells were isolated from the Tunica muscularis of P0-P5, and P60 wnt1-tomato mice using the same procedure described above. After the 30-μm straining step, cells were collected in Hibernate A (Gibco Thermo Fisher Scientific) medium supplemented with N2 supplement (1:100), penicillin (100 U/mL), streptomycin (100 mg/mL), L-glutamine (2 mM), EGF (20 ng/mL), hbFGF (20 ng/mL), and B27 (1:50). FACS was then performed with a BD FACS Aria flow cytometer (BD Biosciences) using a 100-μm nozzle. Forward-sideward scatter dot plots were used to exclude debris and cell aggregates. Endogenous tdTomato was excited by a 488-nm laser. Emission filter was 576/26 nm. For all experiments, purified cells were seeded as outlined above at a concentration of 1.0 × 10^5^ counts/cm^2^.

### FACS of Human LGR-FZD4 Cultures

For FACS analysis, cells were isolated from the Tunica muscularis of human gut specimens as outlined above. After filtration through a 30-μm cell strainer, cells were pelleted at 200 g, resuspended in 20 μL Gamunex (10%-solution, Talecris Biothera-peutics) per 10^6^ cells isolated and incubated for 20 minutes on ice. Then, Fzd4-antibody combined with LGR4 or LGR5 or LGR6 antibody were added in the corresponding concentrations as outlined in Table 3 and incubated for 15 minutes on ice. After antibody incubation, cells were washed 2 times in Hibernate A supplemented with N2 supplement (1:100), penicillin (100 U/mL), streptomycin (100 mg/mL), L-glutamine (2 mM), EGF (20 ng/mL), hbFGF (20 ng/mL), and B27 (1:50). FACS analysis was then performed with a BD FACS Aria flow cytometer using a 100-μm nozzle. Forward-sideward scatter dot plots were used to exclude debris and cell aggregates. Excitation and emission filter settings are described in Table 3. Purified cells were seeded as outlined above at a concentration of 2.0 × 10^5^ counts/cm^2^.

**Table 3:** Antibodies, Biological Samples, Chemicals, Oligonucleotides, Peptides, and Commercial Assays Used in This Study

Reagent/resources	Source	Identifier
Antibodies		
Anti-rabbit IgG (H+L) AlexaFluor488, dilution: 1:400, host: goat	Invitrogen Thermo Fisher Scientific, MA, USA	Cat. No. A-11008
Anti-rabbit IgG (H+L) Alexa Fluor 546, dilution: 1:400, host: goat	Invitrogen Thermo Fisher Scientific, MA, USA	Cat. No. A-11035
Anti-rat IgG (H+L) Alexa Fluor 488, dilution: 1:400, host: goat	Invitrogen Thermo Fisher Scientific, MA, USA	Cat. No. A-11006
Anti-mouse IgG (H+L) Alexa Fluor 488, dilution: 1:400, host: goat	Invitrogen Thermo Fisher Scientific, MA, USA	Cat. No. A-10680
Anti-mouse IgG (H+L) Alexa Fluor 546, dilution: 1:400, host: goat	Invitrogen Thermo Fisher Scientific, MA, USA	Cat. No. A-11030
Anti-mouse IgG (H+L) Alexa Fluor 647, dilution: 1:400, host: goat	Invitrogen Thermo Fisher Scientific, MA, USA	Cat. No. A-21235
BrdU, dilution: 1:100, host: rat	Abcam, Cambridge, UK	Cat. No. ab6326
Fzd4-PE (CD344), dilution: 5μL/10^6^ cells host: mouse, clone: CH3A4A7, Em.: 488 nm, Ex.: 565-605 nm	Bio Legend, CA, USA	Cat. No. 326606
GFAP, dilution: 1:400, host: rabbit	DAKO, Glostrup, Denmark	Cat. No. 0034
HuC/D, dilution: 1:50, host: mouse	Invitrogen Thermo Fisher Scientific, MA, USA	Cat. No. A21271
Ki67, dilution: 1:100, host: mouse	DCS Innovative Diagnostics, Hamburg, Germany	Cat. No. KI68IC002
Ki67, dilution: 1:50, host: mouse	NOVO Castra Leica Biosystems, IL, USA	Cat. No. NCL-MM1
Lgr4-conjugated APC (GPR48), dilution: 10μL/10^6^ cells, host: mouse, clone: #852229, Em.: 620-650 nm, Ex.: 660-670 nm	R&D Systems, Inc., MN, USA	Cat. No. FAB7750P
Lgr4, dilution: 1:50, host: mouse	Invitrogen Thermo Fisher Scientific, MA, USA	Cat. No. SAB370154
Lgr4, dilution: 1:50, host: mouse	Santa Cruz, Biotechnology, TX, USA	Cat. No. sc-390630
Lgr5-conjugated APC, dilution: 10μL/10^6^ cells, host: mouse, clone: #707042, Em.: 620-650 nm, Ex.: 660-670 nm	R&D Systems, Inc., MN, USA	Cat. No. FAB8078A
Lgr5, dilution: 1:50, host: mouse	Invitrogen Thermo Fisher Scientific, MA, USA	Cat. No. MA5-25644
Lgr6-conjugated AF405, dilution: 10μL/10^6^ cells, host: mouse, clone: #918726, Em.: 405 nm, Ex.: 421 nm	R&D Systems, Inc., MN, USA	Cat. No. FAB84581V
Lgr6, dilution: 1:50, host: rabbit	Invitrogen Thermo Fisher Scientific, MA, USA	Cat. No. PA5/109909
P75, dilution: 1:400, host: rabbit	Millipore Merck, Darmstadt, Germany	Cat. No. AB1554
S100β, dilution: 1:100, host: rabbit	Abcam, Cambridge, UK	Cat. No. ab52642
SMA, dilution: 1:50, host: mouse	DAKO, Glostrup, Denmark	Cat. No. M0851
SOX10, dilution: 1:50, host: mouse	Novus Biologicals, CO, USA	Cat. No. NBP2-59050
Beta-3-Tub, dilution: 1:4000, host: rabbit	Bio Legend, CA, USA	Cat. No. 802001
Vimentin, dilution: 1:100, host: rabbit	Abcam, Cambridge, UK	Cat. No. ab45939
Biological samples		
Mice strain C57BL/6J	Jackson Laboratory, Bar Harbor, ME, USA	Stock No. 000664
Mice strain: B6;129S6-Gt(ROSA)26Sortm9(CAG-tdTomato)Hze/J	Jackson Laboratory, Bar Harbor, ME, USA	Stock No. 007914
Mice strain: B6.Cg-Tg(Wnt1-cre)2Sor/J	Jackson Laboratory, Bar Harbor, ME, USA	Stock No. 022501
Human gut samples, Patient-specific data are summarized in Table 2	Department of Pediatric Surgery, University Children’s Hospital Tübingen, Germany	Project Nr. of the local ethical committee 652/2019BO2 and 066/2023BO2
Chemicals, peptides, and recombinant proteins		
Ascorbic Acid-2-phosphate, 1 M	Sigma-Aldrich, Taufkirchen, Germany	Cat. No. A-8960-5G
B27 Supplement, 50x	gibco® Thermo Fisher Scientific, MA, USA	Cat. No. 17504-044
Bovine serum albumin, 100x	Roth, Karlsruhe, Germany	Cat. No. 031166075
BrdU (5-bromo-2’-deoxyuridine), 10 mM	Roche Diagnostics, Mannheim, Germany	Cat. No. 11299964001
Cibrofloxacin Kabi, 2000 μg/mL	Fresenius Kabi, Bad Homburg, Germany	nA
Citric acid monohydrate, 210,14 g/mol	Roth, Karlsruhe, Germany	Cat. No. 5110.2
Collagen-type-I, rat tail, 1 μg/mL	BD Bioscience, Heidelberg, Germany	Cat. No. 354234
Collagenase type XI, 1926 U/mg	Sigma-Aldrich, Taufkirchen, Germany	Cat. No. C9407
DAPI (4’,6-diamidino-2-phenylindolestain), 200 ng/mL	Roth, Karlsruhe, Germany	Cat. No. 6335.1
Dispase type II, 1.01 U/mg	Roche Diagnostics, Mannheim, Germany	Cat. No. D4693
Di-Natriumtetraborat-10-hydrat, 201.22 g/mol	Merck, Darmstadt Germany	Cat. No. 0024611
DMEM (Dulbecco’s modified Eagle’s medium with Ham’s F12 medium 1:1), 1x	Life technologies, Darmstadt, Germany	Cat. No. 21331-020
DNase I, 5%	Sigma-Aldrich, Taufkirchen, Germany	Cat. No. DN25
hEGF (human epidermal growth factor), 40 μg/mL	Sigma-Aldrich, Taufkirchen, Germany	Cat. No. E9644
FCS (fetal calf serum), 100x	Biochrom, Berlin, Germany	Cat. No. S0613
hbFGF (human bone fibroblast-like growth factor), 40 μg/mL	Sigma-Aldrich, Taufkirchen, Germany	Cat. No. F0291
Gamunex, 100 mg/mL	Talecris Biothera-peutics, NY, USA	Cat. No. 80A2828
Goat serum, 100x	Biochrom, Berlin, Germany	Cat. No. 57288
HBSS (Hanks’ balanced salt solution without Ca^2+^ / Mg^2+^), 1x	Sigma-Aldrich, Taufkirchen, Germany	Cat. No. H9394
HBSS (Hanks’ balanced salt solution with Ca^2+^ / Mg^2+^), 1x	Sigma-Aldrich, Taufkirchen, Germany	Cat. No. 55037C
HCl (hydrochloric acid), 2N	VWR® Chemicals, Darmstadt, Germany	Cat. No.: 310701.5000
Hibernate-A	gibco® Thermo Fisher Scientific, MA, USA	Cat. No. A12475-01
Kaiser’s glycerol gelatine	Merck, Darmstadt, Germany	Cat. No. 109242
L-glutamine, 200 mM	Sigma-Aldrich, Taufkirchen, Germany	Cat. No. G7513
Metronidazole, 5000 μg/mL	B. Braun, Melsungen, Germany	nA
N2 supplement, 100x	Life Technologies, Darmstadt, Germany	Cat. No 17502-048
PFA (paraformaldehyde), 30.39 g/mol	Merck KGaA, Darmstadt, Germany	Cat. No. 1.04005.1000
Penicillin/streptomycin, 10.000 U/mL/10 mg/mL	Sigma-Aldrich, Taufkirchen, Germany	Cat. No. P0781
Rock inhibitor, 50 mg	Selleck Chemicals GmbH, Köln, Germany	Cat. No. #688000
R-Spondin1 recombinant mouse, 25 μg	R&D Systems, Inc., MN, USA	Cat. No. 3474-RS
R-Spondin1 recombinant human, 25 μg	R&D Systems, Inc., MN, USA	Cat. No. 4645-RS
Sucrose (D(+)-Saccharose)	PanReac AppliChem, Darmstadt, Germany	Cat. No. A2211.5000
TissueTek	Sakura, Staufen, Germany	Cat. No. 4583
Triton X-100	Roth, Karlsruhe, Germany	Cat. No. 3051.4
WNT3A recombinant mouse, 2 μg	R&D Systems, Inc., MN, USA	Cat. No. 1324-WNT
WNT3A recombinant human, 10 μg	R&D Systems, Inc., MN, USA	Cat. No. 5036-WN
Critical commercial assays		
RNeasy Plus Mini Kit	Qiagen, Hilden, Germany	Cat. No. 74104
QuantiTect Reverse Transcription Kit	Qiagen, Hilden, Germany	Cat. No. 205311
PerfeCTa qPCR ToughMix ROX	Quantabio, Berverly, MA, USA	Cat. No. 733-2093
Oligonucleotides		
Rspo1, mouse fwd 5′-cgacatgaacaaatgcatca-3′ rev 5′-ctcctgacacttggtgcaga-3′	Roche Diagnostics, Mannheim, Germany	Assay ID: 310599
Rspo2, mouse fwd 5′-gtccaggagatgcaagatgg-3′ rev 5′-tcttttgcctttggtgttctc-3′	Roche Diagnostics, Mannheim, Germany	Assay ID: 310663
Rspo3, mouse fwd 5′-tcaaagggagagcgagga-3′ rev 5′-cagaggaggagcttgtttcc-3′	Roche Diagnostics, Mannheim, Germany	Assay ID: 310540
Rspo4, mouse fwd 5′-agagactctgcccaggagaa-3′ rev 5′-ccaacttcctgtccttacgc-3′	Roche Diagnostics, Mannheim, Germany	Assay ID: 310519
Lgr4, mouse	Invitrogen Thermo Fisher Scientific, MA, USA	Mm00554385
Lgr5, mouse	Invitrogen Thermo Fisher Scientific, MA, USA	Mm00438890
Lgr6, mouse	Invitrogen Thermo Fisher Scientific, MA, USA	Mm01291336
Rnf43, mouse	Invitrogen Thermo Fisher Scientific, MA, USA	Mm00552558
Znrf3, mouse	Invitrogen Thermo Fisher Scientific, MA, USA	Mm01191453
Lrp5, mouse fwd 5′-catggacatccaagtgctga-3′ rev 5′-ttgtcctcctcgcatggt-3′	Roche Diagnostics, Mannheim, Germany	Assay ID: 310507
Lrp6, mouse fwd 5′-tcctcgagctctggcact-3′ rev 5′-cctccccactcagtccaata-3′	Roche Diagnostics, Mannheim, Germany	Assay ID: 310507
Gapdh, mouse fwd 5′-agcttgtcatcaacgggaag-3′ rev 5′-tttgatgttagtggggtctcg-3′	Roche Diagnostics, Mannheim, Germany	Assay ID: 307884
Hprt, mouse fwd 5′-tcctcctcagaccgctttt-3′ rev 5′-cctggttcatcatcgctaatc-3′	Roche Diagnostics, Mannheim, Germany	Assay ID: 307879
Tbp, mouse fwd 5′-ggcggtttggctaggttt -3′ rev 5′-gggttatcttcacacaccatga -3′ rev 5′-acccgtgatgggataaacag-3′	Roche Diagnostics, Mannheim, Germany	Assay ID: RN01455648
Software and algorithms		
Axiovision software	Zeiss, Oberkochen, Germany	NA
SigmaStat 3.5 software	Systat Software GmbH, Frankfurt, Germany	NA
Other		
Axio Imager.Z1	Zeiss, Oberkochen, Germany	NA
BD FACS Aria flow cytometer	BD Biosciences, Heidelberg, Germany	NA
Mcllwain tissue chopper	Mickle Laboratory Engineering Co, Guildford, UK).	NA
QIAxcel Advanced	Qiagen, Hilden, Germany	NA
StepOnePlus Real-Time PCR System	Applied Biosystems, Darmstadt, Germany	NA

The medium was supplemented with B27 (1:50) once before seeding. Cells were cultured up to 14 div under proliferation conditions, whereby growth factors (20 ng/mL hEGF/20 ng/mL hbFGF), WNT3A (20 ng/mL, R&D Systems, Inc), R-Spondin1 (100 ng/mL, R&D Systems), as well as Rock Inhibitor (1μM; Selleck Chemicals GmbH) were added daily, and culture medium was exchanged every 5 days. 1 μM BrdU was added after 5 div and 10 div during the proliferation phase.

### Stimulation of Murine and Human Enteric Progenitor Cells

For cell culture experiments, the medium was supplemented either with WNT3A (20 ng/mL), R-Spondin1 (100 ng/mL), or the combination of both after 1 div for murine cultures or on isolation day (0 div) for human cultures. Untreated cells served as control group. To quantify the absolute number of proliferating enterospheres derived from neonate mice and human specimens, brightfield images were taken of stimulated and unstimulated enterospheres at 5 div, respectively, and for human cultures at 14 div.

### Differentiation of Murine and Human Enteric Progenitor Cells

After proliferation phase (5 div for murine and 14 div for human cultures), cell cultures were treated with 10% (v/v) FCS for 2 hours to facilitate attachment of entero- and neurospheres to the culture dishes. Afterwards, differentiation medium (DMEM containing N2 supplement [1:100], penicillin [100 U/mL], streptomycin [100 mg/mL], L-glutamine [2 mM[, 2 % [v/v] FCS, and ascorbic acid-2-phosphate [200 mM; Sigma-Aldrich]) was added and exchanged after 9 div for murine cultures and after 18 div for human cultures. After the differentiation phase (12 div for murine, and 21 div for human cultures), cell cultures were fixed for immunocytochemical analysis.

### Single Sphere Assay

For single sphere assay, cells were isolated from the Tunica muscularis of wnt1-tomato mice aged 2 months (P60), using the same procedure described above for wild-type mice. After FACS sorting, purified cells were seeded as outlined above at a concentration of 2.0 × 10^5^ cells/cm^2^. Cells were cultured up to 7 div under proliferation conditions, whereby growth factors (20 ng/mL hEGF/20 ng/mL hbFGF) were added daily. After 7 div, single neurospheres (40–60 μm diameter) were picked and transferred into a 96-well plate with proliferation medium (DMEM containing N2 supplement [1:100], penicillin [100 U/mL], streptomycin [100 mg/mL], L-glutamine [2 mM], EGF [20 ng/mL], and hbFGF [20 ng/mL]). Before pharmacological stimulation, brightfield images were taken from each single sphere. Afterwards, the medium was supplemented either with WNT3A (20 ng/mL), R-Spondin1 (100 ng/mL), or the combination of both. Single spheres were cultivated under proliferative conditions, whereby growth factors (20 ng/mL hEGF/20 ng/mL hbFGF) were added daily.

To measure diameter changes of stimulated neurospheres, brightfield images were taken after 14 div.

### Time-dependent LGR Expression

For time series experiments, cells were isolated from the Tunica muscularis of wnt1-tomato mice using the same procedure described here for wild-type mice. After FACS sorting, purified cells were seeded as outlined above at a concentration of 1.0 × 10^5^ counts/cm^2^ on collagen type I- (Merck KGaA) coated coverslips and cultivated under proliferative conditions, whereby growth factors (20 ng/mL hEGF/20 ng/mL hbFGF) were added daily. On 5 div, cells were cultivated either under proliferative or differentiation conditions, as already outlined above. On 6 div, 9 div, and 12 div, cells were fixed for immunocytochemical analysis.

### Immunostainings for Differentiation Markers and BrdU Proliferation Assay

After differentiation phase, murine neonate wildtype and human cell cultures were fixed with 4 % (w/v) phosphate buffered paraformaldehyde ([PFA], Merck KGaA) for 20 minutes at room temperature and subsequently rinsed 3 times for 5 minutes in phosphate-buffered saline (PBS). To avoid unspecific binding of antibodies and to permeabilize cells for the detection of intracellular proteins, cell cultures were pretreated with blocking solution containing 4% (v/v) goat serum (Biochrom), 0.1% (w/v) bovine serum albumin ([BSA], Roth), and 0.1% (v/v) Triton X-100 (Roth), for 30 minutes at room temperature. Afterwards, primary antibodies (Table 3) diluted in PBS, 0.1% (w/v) BSA, and 0.1% (v/v) Triton X-100, were added and incubated overnight at 4°C. Afterwards, cell cultures were rinsed with PBS 3 times for 5 minutes. Fluorescent conjugated secondary antibodies (Table 3), diluted in PBS containing 0.1% (w/v) BSA and 0.1% (v/v) Triton X-100, were used for detection of primary antibodies and were incubated for 1 hour, light protected, at room temperature. Nuclear staining was performed together with the secondary antibody, using 4′,6-diamidino-2-phenylindolestain ([DAPI] 200 ng/mL, Roth). For BrdU proliferation assay, cell cultures were pre-treated with 2 N HCl (Roth) in a humidity chamber for 30 minutes at 37°C after the incubation of fluorescent conjugated secondary antibodies. Next, 3 washing steps were carried out, twice with borax buffer (0.1 M (w/v) di-Natriumtetraborat-10-hydrat pH: 8.5, Merck) and once with PBS for 5 minutes followed by incubation of primary BrdU antibody (Table 3) diluted in PBS containing 0.1% BSA, and 0.1% (v/v) Triton X-100, in a humidity chamber for 2 hours at 37°C. Upon 3 washing steps with PBS for 5 minutes, cell cultures were treated with diluted secondary antibody in PBS containing 0.1% (w/v) BSA, and 0.1% (v/v) Triton X-100, together with DAPI (200 ng/mL) for 1 hour at room temperature. Eventually, cell cultures were rinsed again 3 times with PBS for 5 minutes.

### Immunohistochemistry and Ki67 Staining on 5-day-old Enterospheres

To assess proliferation within murine enterospheres during the proliferation phase, 5-day-old enterospheres were picked from the medium and fixed with 4% (w/v) PFA for 20 minutes and rinsed 3 times with PBS. For cryoconservation, fixed enterospheres were stored overnight in 30% (w/v) sucrose solution (Applichem) at 4°C. Afterwards, samples were frozen in isopentane-nitrogen cooled TissueTek (Sakura) and stored at −80°C until further processing. Before staining, cryosections (10 μm) were dried for 1 hour at room temperature, following rehydration with distilled water for 15 minutes.

For paraffin embedding, fixed tissue samples were dehydrated in an ascending alcohol series, followed by xylene and overnight infiltration of paraffin at 60°C. Before staining, paraffin sections (5 μm) were dewaxed by xylene (twice, 5 minutes) and a descending alcohol series (once in 100% ethanol, once in 96% [v/v] ethanol, and once in 70% [v/v] ethanol, 5 minutes each). Finally, slides were rinsed once with distilled water. Next, sections were pretreated with boiled citric acid monohydrate buffer (10 mM, pH 6.0, Merck) for 3 minutes and cooled down at room temperature.

To prevent unspecific binding of antibodies, fixed enterospheres and tissue samples were blocked for 30 minutes with PBS containing 4% (v/v) goat serum, 0.1% (w/v) BSA, and 0.1 % (v/v) Triton X-100, followed by incubation of primary antibodies (Table 3) diluted in PBS with 0.1% (w/v) BSA and 0.1% (v/v) Triton X-100 overnight at 4°C in a humidity chamber. Afterwards, samples were washed with PBS 3 times for 5 to 10 minutes. The secondary antibody (Table 3) was diluted in PBS, 0.1% (v/v) Triton X-100, and 0.1% (w/v) BSA, and incubated for 60 minutes at room temperature. Nuclear staining was carried out with DAPI (200 ng/mL). After 2 washing steps with PBS for 5 minutes, the samples were washed in distilled water for 5 minutes, followed by mounting with Kaiser’s glycerol gelatine (Merck).

### RNA Isolation and RT-PCR

Total RNA of enterospheres was isolated using the RNeasy Plus Mini Kit (Qiagen) according to the manufacturer’s instructions. RNA concentration and integrity were analyzed using QIAxcel Advanced (Qiagen) according to the manufacturer’s instructions. Reverse transcription was carried out with QuantiTect Reverse Transcription Kit (Qiagen). Purified RNA treated without reverse transcriptase served as a negative control. PCR was performed using the StepOnePlus Real-Time PCR System (Applied Biosystems) and the PerfeCTa qPCR ToughMix ROX (Quantabio) according to the manual instructions. The PCR conditions were 50°C for 120 seconds and 95°C for 10 minutes, followed by 40 cycles of 95°C for 15 seconds and 60°C for 60 seconds. The acquisition was performed after the 60°C step of each cycle. Glyceraldehyde 3-phosphate dehydrogenase (GAPDH), hypoxanthine-guanine phosphoribosyltrans-ferase (HPRT), and the TATA binding protein (TBP) were used as reference genes. qRT-PCR was carried out following MIQE quality guidelines. Primers for ligand and receptors are shown in Table 3.

### Microscopy

Images were acquired using a Zeiss Axio Imager.Z1 fluorescence microscope with Apotome module with 358, 488, 543, 647 nm for excitation and appropriate filter sets. Images were acquired using ZEN software, as well as using a the 10-objective (EC Plan-Neofluar 10×/0.30 Ph1), whereby exposure times for DAPI were 500 to 1000 ms, and for neuronal and glial markers 1500 to 2500 ms. Additionally, the 20-objective (Plan-Apochromat 20×/0.8 M27) with exposure times for DAPI <100 ms, Ki67 500 ms, LGR receptors 500 to 1000 ms, and for the neuronal and glial markers, 500 to 1000 ms was used.

### Data Analysis

For analysis of sphere growth, brightfield images were taken after 5 div for murine and after 14 div for human cell cultivation. In total, 7452 murine enterospheres and 4176 human enterospheres were analyzed. The area of spheroids was measured on brightfield images using Axiovision software (Zeiss). Assuming an ideal sphere shape of enterospheres, the theoretical diameter (d) was calculated by equation [1]: $d=2x\sqrt{\frac{A}{\pi }}$ in μm whereby A is the measured area. Further, the total cell volume was calculated by equation [2]: $V=\frac{1}{6}\;x\;\pi d^{3}$ to evaluate a possible pharmacologically effect on the total cell volume of counted enterospheres, whereby V is the obtained volume in μm^3^. It should be mentioned that human progenitors do not form compact spheroids with smooth edges as compared with mouse spheroids; for this reason, we measured the dense compact core. For all, cumulative sphere number as well as volume was normalized to controls to calculate fold change. For each group, 2 technical replicates were analyzed. For the quantification of Ki67^+^/P75^+^/LGR4-6^+^ cells in 5-day-old enterospheres, at least 500 DAPI-positive nuclei with the respective colabeling event were counted to calculate percentages.

To quantify differentiated murine enteric neurons from unpurified cultures, 2 technical replicates per experimental group were counted manually, and mean values were calculated. To evaluate differentiated murine enteric neurons from purified cultures, one technical replicate from small and large intestinal samples per experimental group were counted manually. For the analysis of human differentiation data, 4 technical replicates per experimental group were manually counted, and median value was calculated from 4 biological replicates. qRT-PCR experiments were carried out following MIQE quality guidelines with 6 independent experiments. To evaluate LGR receptor expression in human gut specimens, we applied a semiquantitative evaluation (Table 1). The highest signal intensity for a LGR receptor was found in submucosal neurons expressing LGR6 (ie, high intensity [+++]); thus, we compared each tissue compartment with the signal intensity in those neurons. No expression corresponds to a signal intensity that is comparable to the signal intensity of the negative control (ie, no signal [−]). Low intensity is marked by a dim signal immunoreactivity, which is yet clearly recognizable from the tissue background (ie, low intensity [+]). Staining intensities displaying a strong signal intensity that was still recognizably lower than the immunoreactivity of LGR6 expressing submucosal neurons were defined as medium intensity (++).

### Statistics and Reproducibility

For all experiments, at least 3 independent experiments (independent preparations on different days) were carried out. Statistical analysis was performed using SigmaStat 3.5 software. Results were considered significant at *P* ≤ .05. Statistical analysis for 2 groups was analyzed using Student’s *t*-test or Mann-Whitney rank sum test. Multiple groups were evaluated by either 1-way ANOVA followed by pairwise multiple comparison procedure (Fisher LSD Method), or Kruskal-Wallis 1-way ANOVA on Ranks followed by all pairwise multiple comparison procedure (Student-Newman-Keuls Method). All statistical tests and *P* values are provided in the Supplementary Source Data File.
